# Dopamine receptor antagonists as potential therapeutic agents for ADPKD

**DOI:** 10.1371/journal.pone.0216220

**Published:** 2019-05-06

**Authors:** Parama Paul, Sreekumar Ramachandran, Sheng Xia, Jay R. Unruh, Juliana Conkright-Fincham, Rong Li

**Affiliations:** 1 Center for Cell Dynamics, Department of Cell Biology, Johns Hopkins University School of Medicine, Baltimore, MD, United States; 2 Stowers Institute for Medical Research, Kansas City, MO, United States; 3 Division of Neonatology, Children’s Mercy Hospital, Kansas City, MO, United States; 4 Department of Chemical and Biomolecular Engineering, Whiting School of Engineering, Johns Hopkins University, Baltimore, MD, United States; University of Sheffield, UNITED KINGDOM

## Abstract

Autosomal dominant polycystic kidney disease (ADPKD) is caused mostly by mutations in polycystin-1 or polycystin-2. Fluid flow leads to polycystin-dependent calcium influx and nuclear export of histone deacetylase 5 (HDAC5), which facilitates the maintenance of renal epithelial architecture by de-repression of MEF2C target genes. Here, we screened a small-molecule library to find drugs that promotes nuclear export of HDAC5. We found that dopamine receptor antagonists, domperidone and loxapine succinate, stimulate export of HDAC5, even in *Pkd1*^*–/–*^cells. Domperidone targets Drd3 receptor to modulate the phosphorylation of HDAC5. Domperidone treatment increases HDAC5 phosphorylation likely by reducing protein phosphatase 2A (PP2A) activity, thus shifting the equilibrium towards HDAC5-P and export from the nucleus. Treating *Pkd1*^*–/–*^mice with domperidone showed significantly reduced cystic growth and cell proliferation. Further, treated mice displayed a reduction in glomerular cyst and increased body weight and activity. These results suggest that HDAC5 nucleocytoplasmic shuttling may be modulated to impede disease progression in ADPKD and uncovers an unexpected role for a class of dopamine receptors in renal epithelial morphogenesis.

## Introduction

ADPKD is one of the most common and life-threatening genetic diseases leading to end-stage renal failure, yet to date there are few therapeutic interventions against this disease. Most cases of ADPKD are caused by heterozygous germline mutations in either the *Pkd1* or *Pkd2* gene [1]. *Pkd1* encodes for polycystin-1 (PC1) protein and *Pkd2* encodes for polycystin-2 (PC2) protein. PC1 and PC2 interact via their C-terminal tails to form a receptor-calcium channel complex, which some have proposed to sense mechanical stress exerted on renal epithelial cells [2–4]. Mouse with homozygous deletion of *Pkd1* die *in utero* between embryonic day 14.4–15.5 ([5]. Mouse models of conditional *Pkd1* gene disruption showed that loss of *Pkd1* at 14 days after birth does not cause immediate polycystic phenotype. Renal injury drastically accelerates cyst formation, suggesting that polycystins either play a protective role against stress-induced injury or orchestrate proper repair of damaged tissue. Cystic growth is driven by a combination of abnormal proliferation of cyst lining cells and transepithelial fluid secretion into cyst lumen, a process driven by intracellular 3', 5'- cyclic adenosine monophosphate (cAMP) via apical cystic fibrosis transmembrane conductance regulator (CFTR) Cl^-^ channel [6–11]. Thus, a potential avenue for treating ADPKD may reside in therapeutic restoration of the protective functions disrupted by polycystin mutations.

Our previous work demonstrated that a cellular response to polycystin and fluid flow-induced intra-cellular calcium rise in nuclear export of HDAC5 and concomitant activation of MEF2C transcriptional targets [12]. HDAC5 is a Class IIa HDAC that contains both NLS (nuclear localization signal) and NES (nuclear export signal) and shuttles between the nucleus and cytoplasm [13, 14]. In the nucleus, these HDAC proteins associate with various transcription factors and corepressors to silence the transcription of various genes [15, 16]. Extracellular stimuli, including mechanical stress, can regulate the nuclear export of class II HDACs by activating specific kinases, such as calcium/calmodulin-dependent protein kinase [17] and protein kinase C [12, 18], which phosphorylate class IIa HDACs at different serine residues. This phosphorylation leads to the recruitment and binding of 14-3-3 [19]. Nuclear export following binding of 14-3-3 results from masking of NLS sequence [19] or exposing of NES sequences, via conformational change [17].

Class IIa HDACs are signal-responsive regulators of gene expression in various systems such as cardiac hypertrophy, regulation of angiogenesis, and neuronal differentiation [20–24]. Following stress or injury, phosphorylation and nuclear export of these HDACs enables the reactivation of the developmental transcriptional program mediated by the MEF2 family transcriptional activators [20]. Our previous work found that HDAC5 specifically responds to fluid sheer stress signal via polycystins, allowing transcriptional activation of MEF2C target genes, many of which are likely to be involved in epithelial differentiation and morphogenesis [12]. By contrast, *Pkd1*^*–/–*^collecting-duct epithelial cells are deficient in stress-induced export of HDAC5, which is likely to contribute to the diminished epithelial integrity and repair ability of the mutant renal tissue. Thus, agents that promote the nuclear export of HDAC5 from the *Pkd1*^*–/–*^cell nucleus and, thus, de-repress MEF2C target genes should have therapeutic potential for treating ADPKD. In this study, we first show genetic inactivation of *Hdac5* can suppress cyst formation in *Pkd1*^*–/–*^mice. We then sought to find compounds that can cause nuclear export of HDAC5 in a flow-independent manner. We performed a screen using a FDA-approved compound library and identified dopamine receptor antagonists as potent promoters of HDAC5 export in renal epithelial cells bypassing the requirement for both PC1 and fluid flow stimulation. We further investigated the mechanism by which dopamine receptor antagonists promotes HDAC5 nuclear export and demonstrated the ability of one of these compounds to slow cyst growth in *Pkd1*^*–/–*^mice.

## Materials and methods

### Antibodies and chemicals used

HDAC5 antibody was obtained from Cell Signaling (Catalog #2082), HDAC5 (Phospho-Ser498) antibody was obtained from Signalway (Catalog #11193–2), 14-3-3 antibody from Santa Cruz Biotechnology (Catalog # sc-1657) and fluorescein labeled Dolichos Biflorus Agglutinin from Vector Laboratories (Catalog # FL-1031). SCH 23390 hydrochloride, tautomycetin was from R&D systems. IBMX, Sp-5,6-DCI-cBiMPS, H-89 Dihydrochloride, okadaic acid and 8-Bromo-cAMP are from Santa Cruz Biotechnology. Haloperidol hydrochloride, SCH 202676 hydrobromide and SQ 22536 are from Tocris. Tetrahydrozolone hydrochloride, lofexidine hydrochloride and methyldopate hydrochloride 200 were from VWR. The rest of the chemicals were purchased from Sigma.

### Small-molecule screening

HDAC5-GFP *Pkd1*^*loxP/loxP*^ cells were screened against 1200 compounds in the Prestwick Chemical Library (Prestwick Chemical, Illkirch, France) to determine the effect of these compounds on HDAC5-GFP localization within the cells. HDAC5-GFP *PKD1*^*loxP/loxP*^ cells were plated at a concentration of 8000 cells/well in 40 μl of culture medium into 384well CellCarrier imaging plates (PerkinElmer, Waltham, MA) using a Biotek Multiflo automated dispenser (Biotek, Winooski, VT); then incubated for 72 hours at 33°C and 5% CO_2_. Compounds, PMA & I3A positive controls or DMSO negative controls (400 nl) were transferred from 100 μM or 10 μM stock plates using a Platemate Plus automated pipetting station fitted with a positive displacement steel needle syringe head (Matrix Technologies, Hudson, NH) onto the plated cells for a final concentration of 1 μM or 100 nM, respectively. Plates (n = 2 for each stock plate and final concentration) were incubated for 90 min at 33°C and 5% CO_2_ prior to fixing the cells by adding 40 μl of prewarmed culture medium containing 8% paraformaldehyde and 2% TritonX-100 for 20 min at room temperature. Imaging plates were stained on a Tecan Freedom Evo 200 liquid handling robotic platform (Tecan, Mannedorf, Switzerland) with an integrated Biotek EL406 plate washer/dispenser (Biotek). Briefly, fixation medium was aspirated and the plates were washed with 2 x 80 μl PBS. Cell nuclei were stained by pipetting 50 μl staining solution containing 1 μg/ml DAPI in PBS and incubated 15 min at room temperature. Plates were washed with 3 x 80 μl PBS; then 50 μl of PBS was dispensed into the plates. The plates were spun down at 1000 rpm for 5 min to remove any bubbles and sealed with clear imaging seals. Cells were imaged for HDAC5-GFP fluorescence and DAPI-stained nuclei with an Operetta high content imager (PerkinElmer) using 10x objective (3 spots per well).

### Screen image analysis

All image processing was done in the open source software package, ImageJ using custom plugins available at http://research.stowers.org/imagejplugins. First, DAPI images were thresholded with the default ImageJ autothreshold (IsoData) method. Nuclei adjacent to the image border as well as nuclei with area under 4 pixels or over 1000000 pixels were eliminated. The cytoplasmic region was estimated as a 4 iteration dilation of the nuclear region, where closely spaced nuclei were not allowed to merge. The minimum intensity of the GFP image (ignoring dead pixels) was subtracted from each image as an estimate of background intensity. Tests with more complex methods like rolling ball background subtraction did not change the resulting statistics significantly. Then the average intensity of each nucleus and associated cytoplasmic region were measured and the nuclear/cytoplasmic ratio was calculated.

All pilot screens with DMSO showed a biphasic response with nuclear enriched (nuclear/cytoplasmic ratio ~3) and unenriched (nuclear/cytoplasmic ratio ~1) populations. On a logarithmic scale, the midpoint between these populations is 1.2. Therefore, 1.2 or in other words 20% above the cytoplasmic value was set as a threshold for “nuclear” classified cells. For each screen image, the fraction of nuclear cells was calculated along with the standard error in the mean for the 3 images. 28 control DMSO wells were scattered on each plate. They did not show a spatial or plate dependence, so they were pooled for hit analysis. Wells were considered hits if the fraction nuclear was more than 3 standard deviations different from the DMSO distribution center. Given that the screen was run twice, hits were listed in S1 and S2 Tables if they were hits in either run.

P values for hits were calculated as follows. The average fraction nuclear and its standard error were calculated from all six images (three from each run). The t distribution was then used to calculate the probability that these values were greater than or equal to the DMSO average for positive hits (nuclear export) and less than or equal to the DMSO average for negative hits (nuclear enrichment).

### Lentiviral knockdown cell lines

*PKD1*^*loxP/loxP*^ cells were individually transfected with Drd1a (Cat # iV046437), Drd2 (Cat # iV043550) and Drd3 (Cat # iV037040) pooled RNAi lentivirus (Applied Biological Materials Inc.) following the standard protocol and stable lines were generated by puromycin selection. Then these cells were treated with drug and harvested for biochemical analysis.

### Mouse strains and cell line preparation

All mouse strains used in this study were of the C57BL/6 background. *Pkd1*^*loxP/loxP*^ mice (The Jackson laboratory) and tamoxifen-*Cre* (*EsrCre*) mice (Stowers Institute) were crossed to generate *Pkd1*^*loxP/+*^ *EsrCre* mice, which were then paired to generate *Pkd1*^*loxP/loxP*^ *EsrCre* (which is referred as *Pkd1*^*loxP/loxP*^) mice. Then *Pkd1*^*loxP/loxP*^ mice were paired with *Hdac5* ^*–/–*^mice (from E. Olson, University of Texas Southwestern Medical Center) [25] to obtain *Pkd1*^*loxP/loxP*^ *Hdac5* ^*+/–*^.

For cell line generation we crossed *Pkd1*^*loxP/loxP*^ *EsrCre* with immortal (IM) mice carrying SV40 large T antigen (Linheng Li, Stowers Institute) to obtain *Pkd1*^*loxP/-*^ *EsrCre IM* male mice, which then were paired with *Pkd1*^*loxP/loxP*^ *EsrCre IM* female to generate *Pkd1*^*loxP/loxP*^ *EsrCre IM* mice. Kidneys were collected when *Pkd1*^*loxP/loxP*^ *EsrCre IM* mice were 2 months old. Cells were dissociated with 0.5% collagenase IV and plated in DMEM containing 10% fetal bovine serum at 37°C for three days. The cells were treated with 1mg/L tamoxifen for two days. After trypsinization, cells were incubated with DBA-FITC (Vector Lab) and single cell sorting into 96-well plates was carried out. Genotyping was done to select *Pkd1*^*–/–*^ colonies. We also followed the same protocol, except tamoxifen treatment, to get the *Pkd1* ^*loxP /loxP*^ cells. Similarly, we prepared cells from *Kif3a* ^*loxP/loxP*^ *EsrCre* and *Hdac5* ^*–/–*^mice. *Kif3a* ^*loxP/loxP*^ mice were originally provided by P. Igarashi (University of Texas Southwestern Medical Center) [26].

### Cell culture and drug treatment

*PKD1*^*loxP/loxP*^ cells were transfected with plasmid containing C-terminally GFP tagged HDAC5 using LipoD293^TM^ transfection reagent from SignaGen Laboratories following the standard protocol. Cells that stably express the transfected DNA were then selected. Single cell sorting of GFP expressing cells using a flow cytometer MoFlo legacy was performed to get monoclones. For sorting, we first trysinized the cells and resuspended the cell pellet in 500 μl culture medium. Then, the cells were stained with 7-Aminoactinomycin D (7-AAD), a cell viability dye, for 10 minutes. Single viable GFP-positive cell was added to each well of 96 well plate. The cells were then cultured at 33°C. Before using these cells, they were checked for proper localization (nuclear) and flow response [27]. Cells were grown to confluence in culture medium (DMEM containing 2% fetal bovine serum, 0.75μg/l interferon-γ, 1g/l insulin, 0.67 mg/l sodium selenite, 0.55g/l transferrin, 0.2g/l ethanolamine, 36ng/ml hydrocortisone, 0.1μM 2,3,5-triido-L-thyronine, 100 units penicillin-G, 0.3 mg/ml glutamine and 100μg streptomycin sulfate) at 33°C and 5% CO_2._ The cells were treated with drugs for 90 min and fixed using 4% paraformaldehyde.

### Negative hit validation

HDAC5-GFP expressing *PKD1*^*loxP/loxP*^ cells were grown to confluence. Culture media with DMSO or terbutaline hemisulfate was run at 0.2 ml/min for 30 minutes over the cells using a peristaltic pump to induce fluid shear stress [12] and then HDAC5-GFP localization was quantified. Confluent HDAC5-GFP *PKD1*^*loxP/loxP*^ cells were also treated with a PKC activator, PMA, in addition to DMSO or terbutaline hemisulfate. Following incubation for 30 minutes the cells are fixed with 4% PFA and HDAC5-GFP localization is quantified.

### Nuclear and cytoplasmic fractionation

Fractionation was done using protocol as in [28] with some modification. All the buffers were kept on ice and centrifugation was done at 4°C using low brake. Protease inhibitor and 1 mM DTT was added to all buffers before use. *PKD1*^*loxP/loxP*^ cells were grown in 15 cm tissue culture until they became confluent. Then they were treated with either domperidone, loxapine succinate or DMSO for 90 minutes. Following incubation with drug the cells were washed with PBS twice and were harvested by scraping and centrifugation. The cell pellet was resuspended in two times the volume of pellet with buffer A (10 mM HEPES, pH 7.9, 1.5 mM MgCl_2_, 10 mM KCl, 0.5 mM DTT) and incubated on ice for 15min, followed by homogenization using loose dounce homogenizer. Cell lysis was checked by trypan blue staining of the nucleus after every 20 strokes. Cell lysate was then centrifuged at 25,000g for 20 minutes. The supernatant will give the S100 fraction and the pellet will give the nuclear fraction. The supernatant is transferred to a fresh tube and 0.11 times volume of buffer B (0.3 M HEPES, pH 7.9, 30 mM MgCl_2_, 1.4 M KCl) is added. This is centrifuged at 40,000 rpm in a 70.1Ti Beckman rotor for 45 minutes. The supernatant is the S100 fraction. The nuclear pellet from the previous step was resuspended in buffer C (20 mM HEPES, pH 7.9, 25% glycerol, 1.5 mM MgCl_2_, 0.2 mM EDTA, 0.5 mM DTT). NaCl was added and rotated for 30 minutes at 4°C to lyse the nucleus. This is centrifuged at 40,000 rpm in a 70.1Ti Beckman rotor for 45 minutes. The supernatant is the nuclear fraction.

### Drug treatment of mice

*Pkd1*^*loxP/loxP*^ *EsrCre* mice were crossed. Cre recombinase activity was induced in mice by intraperitoneally injecting nursing mothers with tamoxifen (200 mg/kg) in corn oil (Sigma-Aldrich) when their pups were at P10 & P11. From P12-P20 the mice received intraperitoneal injection of 10 mg/kg domperidone [29, 30]. At the end of this treatment the animals were sacrificed and kidneys were dissected out for biochemical and histological examination. For the P12-P42 treatment group, pups received IP injection of 10mg/kg domperidone until P42 and were sacrificed on P43. The kidneys were fixed in 4% paraformaldehyde and embedded in paraffin. 5 μm thick sections were cut and Hematoxylin and eosin stainin (H&E) staining was done. The H&E stained kidney sections were imaged using Axiovert 206 widefield microscope and cystic area & number of cysts was quantified using Image J. The fraction cystic area was quantified by first smoothing the image in ImageJ with a Gaussian blur with 1pixel standard deviation. The tissue containing region was thresholded by selecting all pixels below 87% of the difference between the background and the minimum intensity in green and blue channels. A boundary around each kidney section was drawn manually and regions outside of it were set to zero. The holes in this mask are the cysts and were selected for further processing. Holes within the cysts were filled. Next cysts smaller than 400 pixels were eliminated. Finally, cyst regions were manually curated to correct mistakes in the automated selection. Finally, the kidney area was determined from the manual outline and cyst area was summed for each section to calculate the fraction cystic area. Fraction cystic area was multiplied by 100 to give the percentage cystic area.

The sections were also stained with Ki67 antibody (Thermo scientific, Catalog # 9106–5) with hematoxylin counterstain. ImageJ was used to quantify the number of Ki67 positive nuclei (brown nuclei) and total number of nuclei. For this quantification, the images were first converted to absorbance units by assuming that the background regions represented 100% transmission and black regions represent 0% transmission. Next the images were spectrally unmixed (three spectral channels: r, g, and b) using Ki67 positive and negative nuclei as reference. While this does not achieve perfect separation of Ki67 positive and negative signals, it increases the signal/background to the point where automatic quantification is straightforward. Finally, the number of maxima in either unmixed channel were determined by repeatedly finding the maximum intensity above a threshold (set at 20% of the maximum intensity in each channel) and clearing the 9 pixel diameter area around that point. This was repeated until no pixels above threshold were found.

### Blood urea nitrogen (BUN) measurement

For the *Pkd1*^*–/–*^*Hdac5* study, the blood was collected in BD vacutainer PST^TM^ Hemograd^TM^ tubes coated with lithium heparin (BD Vacutainer, Catalog # 367960). The plasma was separated by spinning and collected the supernatant in a separate tube. The BUN was measured using the QuantiChrom^TM^ Urea Assay kit (BioAssay Systems, DIUR-500).

### Statistics

The percentage cystic area, fraction of Ki67 nuclei and BUN values were normalized to the respective litter’s DMSO treated mouse or *Pkd1*^*-/-*^ *Hdac5*^*+/+*^ in order to avoid litter to litter variation in cyst formation. Data are presented as mean ±SEM. Statistical significance between two groups was tested by 2-tailed Student’s *t* test. Values above 0.05 was considered significant.

### Study approval

The Institutional Animal Care and Use Committee of the Johns Hopkins University School of Medicine and of Stowers Institute for Medical research approved all experiments involving mice in this study. Mice were housed with free access to food and water under a 12 hour light/12 hour dark cycle. Mice were fed a diet containing low fiber (5%), protein (20%) and fat (5–10%). Mouse rooms were maintained at 30–70% relative humidity and a temperature of 18–26°C (64–79°F) with at least 10 room air changes per hour. Euthanasia in mice was performed by carbon dioxide asphyxiation, followed by cervical dislocation. All experiments were performed in accordance with relevant guidelines and regulations.

## Results

### Genetic inactivation of Hdac5 in *Pkd1* ^*–/–*^mice suppresses cyst formation

Our previous study found that *Hdac5* heterozygosity and chemical inhibition by pan-HDAC inhibitor, trichostatin A, reduced cyst formation in *Pkd2* ^*–/–*^mouse embryos [12]. In this work, we first wanted to test whether genetic inhibition of *Hdac5* could also reduce cyst formation in *Pkd1*^*–/–*^mice after birth. Pairs of *Pkd1* ^*loxP /loxP*^ *Hdac5*^*+/–*^*EsrCre* mice were crossed to obtain *Pkd1* ^*loxP /loxP*^ *Hdac5*^*+/+*^, *Pkd1* ^*loxP /loxP*^ *Hdac5*^*+/–*^and *Pkd1* ^*loxP /loxP*^ *Hdac5* ^*–/–*^in littermates. *Pkd1* was inactivated by tamoxifen injection at postnatal day 10 and 11 (P10, P11), within the critical developmental period for cyst formation [31] and kidneys were dissected at P21 and P28. *Pkd1*^*–/–*^*Hdac5*^*+/–*^kidneys had reduced cystic area percentage compared to *Pkd1*^*–/–*^*Hdac5*^*+/+*^ (S1A–S1C Fig). Renal function, as measured by the blood urea nitrogen (BUN) level from blood plasma, was not improved comparing P21 pups, at a later time, P28, *Hdac5* heterozygous pups did show significant improvement (S1D Fig). Even though, the cystic area and BUN for double mutants (*Pkd1*^*–/–*^*Hdac5*^*-/-*^) was further reduced, we did not have enough samples to perform statistical analysis, due to very few viable double mutants in each litter. These results reconfirm the finding of our previous study in *Pkd2* ^*–/–*^mouse embryos. It shows that *Hdac5* inhibition reduces renal cyst formation and improves renal function in an adult ADPKD model.

### Small-molecule screen to identify flow-independent regulators of HDAC5 nuclear export

Repression of transcription by HDAC5 depends on its localization in the nucleus [19], and *Pkd1* null cells are deficient in HDAC5 nuclear export in response to fluid flow stimulation. To better understand the signaling network that regulate HDAC5 nuclear export and to search for mechanisms that could rescue the defects of *Pkd1* mutant cells, we performed a small molecule screen, using a library of mostly FDA-approved and well characterized compounds to identify those that could induce HDAC5 export in the absence of fluid flow stimulation. To this end, we produced a *Dolichos biflorus* agglutinin (DBA)-positive collecting duct cell line from *Pkd1* ^*loxP /loxP*^ SV40 large T antigen mice (see methods for details, S5 Fig) and stably expressed HDAC5-GFP fusion protein. A high content imaging-based platform was used to perform the screen. The compound concentration and incubation time was based on the best working condition of the positive control phorbol 12-myristate (PMA) [12, 18, 32]. Pilot runs were performed to optimize the number of cells to be seeded to obtain a polarized monolayer within 72 h. The polarized epithelial cells were treated with compounds at two different concentrations, 100nM and 1μM, for 90 min (S2A Fig). The cells were subsequently fixed, nuclei were stained with 4',6-diamidino-2-phenylindole (DAPI), and the images were subjected to automated analysis of nuclear/cytosolic ratio of HDAC5-GFP using a custom software (see Methods). Using a stringent cutoff of three standard deviation difference from DMSO, we obtained 70 hit compounds (5.8% hit rate, referred to hereafter as positive hits, S1 Table) that showed significant export of HDAC5 compared to negative control (DMSO). For unknown reasons, when the high-throughput screening was performed, we could see that there was a low level of HDAC5 export in the negative control (DMSO), though the fluorescence was still higher in the nucleus. This allowed us to identify compounds that enhanced nuclear accumulation of HDAC5 compared to the negative control (referred to hereafter as negative hits, 27 hit compounds, 2.2% hit rate, S2 Table).

Interestingly, 40% of the positive hits were G-protein coupled receptor (GPCR) ligands and 75% out of these were receptor antagonists (S2C Fig). On the other hand, 70% of the negative hits were GPCR ligands and 95% of these were receptor agonists (S2D Fig). Although the library itself is enriched for GPCR targets, this trend probably indicates a common mechanism, whereby, antagonism of GPCR promotes HDAC5 nuclear export, however, stimulation of GPCR promotes HDAC5 nuclear accumulation. We, therefore, went on to validate the GPCR ligands in the hit list. In this functional category, we chose 7 of the positive hit compounds (Cisapride, Loxapine succinate, Oxybutynin chloride, Domperidone, Pimozide, Brinzolamide and Beta-Escin) and 3 of negative hit compounds (Terbutaline hemisulfate, Formoterol fumarate and Isoetharine mesylate salt) to validate based on analysis of the literature. Among these, the once that caused HDAC5 export were domperidone and loxapine succinate, two dopamine receptor antagonists (S2B Fig). The negative hit validated was terbutaline hemisulfate, a β_2_-adrenergic receptor agonist, which caused nuclear import of HDAC5 following fluid-flow mediated and PMA-mediated export of HDAC5 (S3A and S3C Fig).

### Dopamine receptor antagonists cause HDAC5 nuclear export in a cilia- and *Pkd1*-independent manner

**Fig 1:**
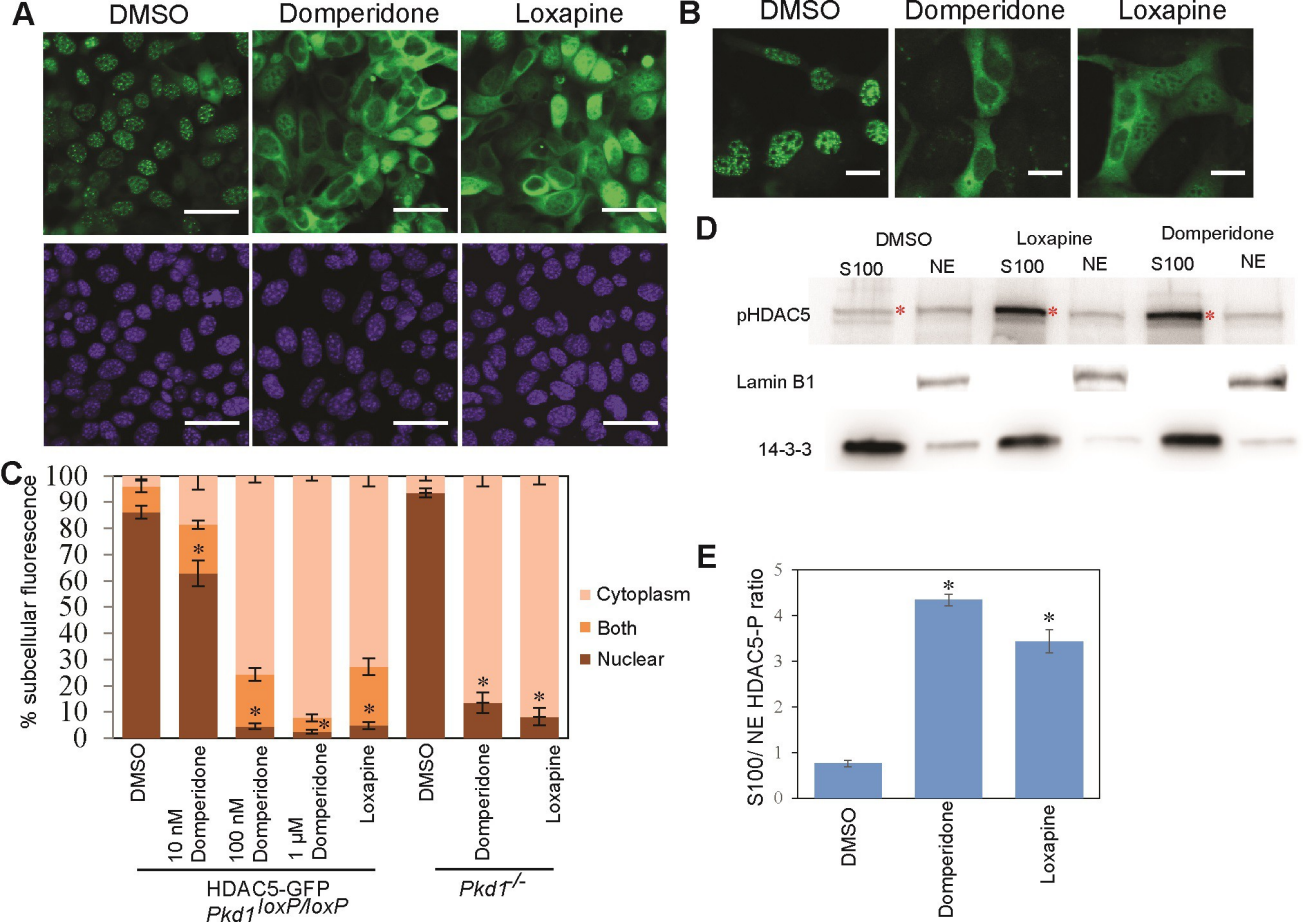
Dopamine antagonists stimulate nuclear export of HDAC5. (A) HDAC5-GFP-expressing *PKD1*^*loxP/loxP*^ cells were subjected to treatment with DMSO, 1 μM domperidone or 1 μM loxapine succinate, as indicated. Representative images show that HDAC5 is exported out of the nucleus marked by DAPI (blue) following treatment with the drugs but not in DMSO. Scale bars: 20 μm. (B) The same treatments as above was applied to *Pkd1*^*–/–*^cells showing that domperidone and loxapine succinate cause nuclear export of HDAC5 in a PKD1-independent manner. Scale bars: 20 μm. (C) Quantification of percentage of cells with HDAC5-GFP in the cytoplasm, nucleus, or both compartments. Bar graphs show averages from > 3 independent experiments (>150 cells counted per experiment). Error bars: standard error of the mean (SEM). * indicate P value < 1x 10^−5^ for nuclear population compared to the respective DMSO control. (D) *PKD1*^*loxP/loxP*^ cells were treated with DMSO, loxapine succinate or domperidone and nuclear (NE) and cytoplasmic (S100) fractions were separated. Immunoblot analysis of HDAC5 phosphorylation using an antibody against phosphorylated serine 489, and control antibodies as indicated, was performed. * indicate the correct band of pHDAC5. (E) Quantification of the western showing increased HDAC5 phosphorylation ratio following drug treatment. Error bars: standard error of the mean (SEM). * indicate P value <0.01.

Domperidone (trade names Motilium, Molax etc.) is a peripheral dopamine receptor antagonist that does not cross the blood-brain barrier. It is used to relieve vomiting and nausea and treat reflux gastritis [33, 34]. Loxapine succinate (trade names Loxapac, Loxitane) is another dopamine receptor antagonist commonly used as an antipsychotic medication [35]. Both drugs potently caused the export of HDAC5-GFP from the nucleus in both *PKD1*^*loxP/loxP*^ cells stably or transiently transfected with HDAC5-GFP (Figs 1A and 1C and S3B and S3C). This domperidone mediated HDAC5 export is dose dependent (Fig 1C). To confirm the effect of the drugs on endogenous HDAC5, we performed fractionation experiments to separate nuclear (NE) and cytosolic (S100) fractions. Immunoblotting using an antibody against phosphorylated Ser489 site (equivalent to Ser498 of human HDAC5) showed that nuclear export caused by these drugs was associated with Ser489 phosphorylation, consistent with the previously identified mechanism regulating HDAC5 nuclear export (Fig 1D and 1E).

*Pkd1*^*–/–*^cells are impaired in fluid-flow sensing in renal epithelium cells [12, 36] and also in sensing vascular endothelial stress [37]. To test if domperidone and loxapine succinate bypass the requirement for PC1 in fluid flow-induced HDAC5 export, we transiently transfected *Pkd1*^*–/–*^DBA cells with HDAC5-GFP and performed drug treatment. Both domperidone and loxapine succinate caused export of HDAC5 in the *Pkd1*^*–/–*^cells similar in level to that in *PKD1*^*loxP/loxP*^ cells (Fig 1B and 1C). The mechanosensory function of the renal epithelium is attributed to the primary cilium, which is independent of the cilia specific calcium influx [38]. Dopamine receptors are known to be localized to the primary cilium [39, 40], along with its other localization. We tested if export of HDAC5 caused by domperidone and loxapine succinate was dependent on the presence of cilia. Knockout of *Kif3a*, encoding a subunit of kinesin-II, is known to disrupt cilia formation in renal epithelial cells [26]. We confirmed that *Kif3a*^*–/–*^DBA cells cultured from the corresponding mutant mice were indeed devoid of cilia (S4A and S4B Fig). However, both drugs caused robust export of HDAC5 in *Kif3a*^*–/–*^cells transiently transfected with HDAC5-GFP (S4C and S4D Fig), suggesting that the effect of these drugs was independent of the cilia, thus revalidating that this pathway is independent of flow and the ciliary localization of dopamine receptor is not crucial for this response.

### Dopamine receptor antagonists likely work via Drd3 receptor

**Fig 2:**
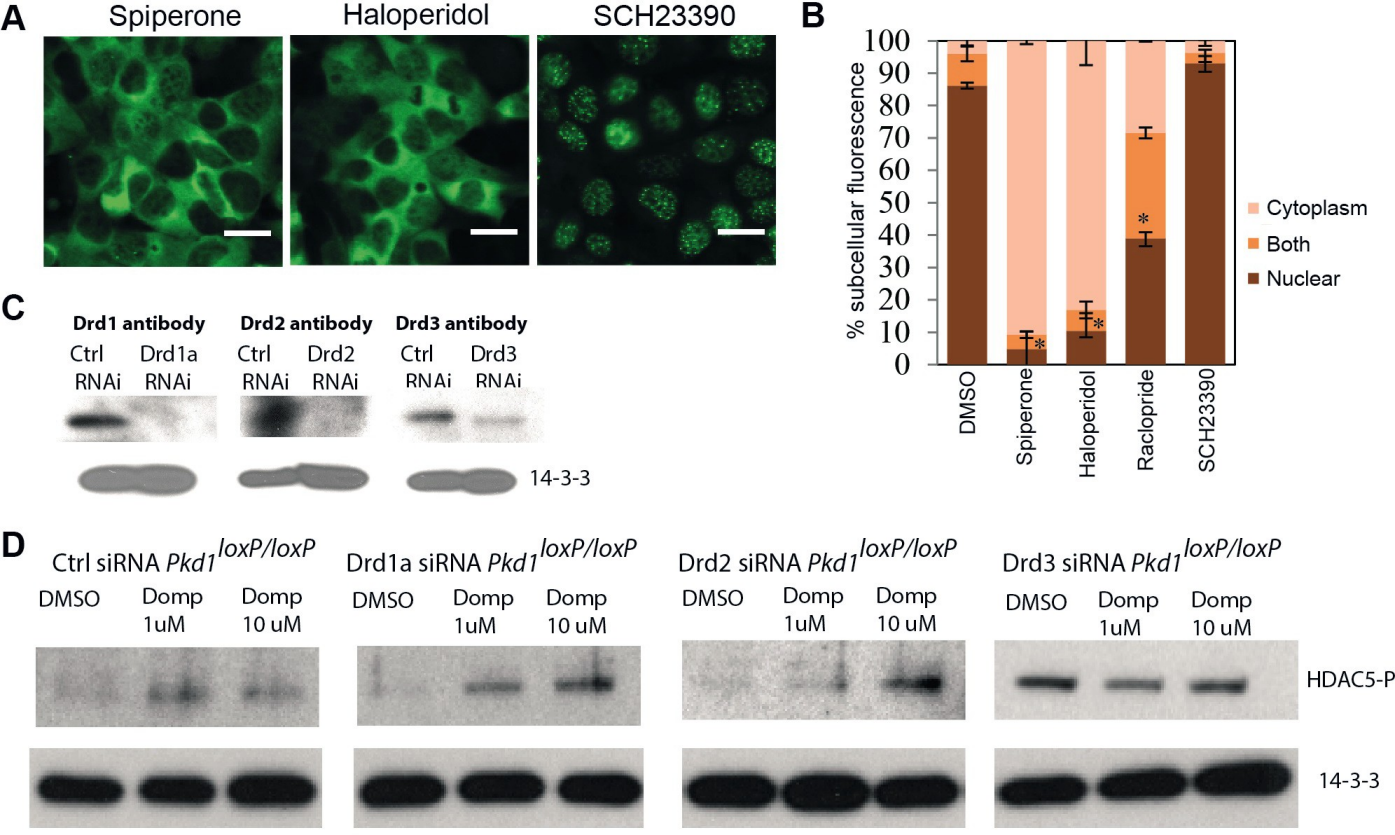
Drug-stimulated nuclear export of HDAC5 is linked to Drd3 receptor antagonism. HDAC5-GFP-expressing *PKD1*^*loxP/loxP*^ cells were treated with dopamine receptor antagonist that are specific towards different dopamine receptor subtypes. (A) Representative images show that HDAC5 is exported from the nucleus by DA_2_ specific antagonists, spiperone (30 mM) and haloperidol (100 mM), but not by DA_1_ specific antagonist, SCH23390 (300 mM). Also, Drd2/Drd3 (members of DA_2_ receptor subtype) specific antagonist, Raclopride (1 mM) can cause export of HDAC5. (B) The plot shows the quantification of percentage of cells with HDAC5-GFP localized in the cytoplasmic, nuclear compartment or both. Each bar is an average of >3 independent experiment (>140 cells counted per experiment). Error bars: SEM. Scale bars: 20 mm.* indicate P value < 7x10^-6^ for nuclear population compared to DMSO control. (C) Western shows that Drd1a, Drd2 and Drd3 pooled siRNA lentivirus cause significant knockdown of respective receptors in stably transfected *PKD1*^*loxP/loxP*^ cells. The primary antibody used for western is mentioned over each blot. (D) Western showing the loss of dose-dependent HDAC5-P increase in Drd3 knockdown cells, compared to control (Ctrl) or Drd1a, Drd2 siRNA cell lines.

Dopamine receptors are classified into two major families, dopamine -1 (DA_1_)-like and dopamine-2 (DA_2_)–like receptors. Involvement of the DA_1_ receptor in the natriuretic/diuretic and vasodilator function in the kidney has been well studied [41, 42], however, involvement of the DA_2_ receptor in kidney function is poorly understood. Studies in dogs showed that DA_2_ receptors play a role in renal function [43]. To investigate whether HDAC5 export, in response to dopamine antagonist, is carried out by DA_1_ or DA_2_ receptor subtype, we tested other known antagonists that are specific for either DA_1_ or DA_2_ or both. Spiperone, a known DA_2_-specific antagonist [44], caused nuclear export of HDAC5, as did Haloperidol, an antagonist for DA_1_ & DA_2_ [44] (Fig 2A and 2B). Raclopride, a more specific D2/D3 antagonist, can cause nuclear export of HDAC5 (Figs 2B and S4D). SCH23390, an antagonist specific for DA_1_ [44], did not cause nuclear export of HDAC5 (Fig 2A and 2B). These data suggest that HDAC5 export is due to antagonism of DA_2_ receptor subtype, not DA_1_ receptor subtype. This unravels a dopamine receptor type-specific regulation of HDAC5 shuttling in renal epithelial cells.

In mouse, DA_1_ receptor family includes Drd1 and Drd5 and DA_2_ receptor family includes Drd2, Drd3 and Drd4. Among these Drd1a, Drd2 and Drd3 are expressed at appreciable level in mouse kidneys (our unpublished transcriptomic data). We used pooled siRNA lentivirus to knockdown Drd1a, Drd2 and Drd3 in *PKD1*^*loxP/loxP*^ cells (Fig 2C). Only knockdown of Drd3 was able to abolish the dose-dependent increase in HDAC5 phosphorylation (Fig 2D), compared to control, Drd1a and Drd2 knockdown cells. Further, the knockdown of Drd3 mimics the receptor antagonism in causing HDAC5 phosphorylation in DMSO treated cells (Fig 2D). These data suggest that HDAC5 export is due to antagonism of Drd3, a DA_2_ receptor family member.

### Dopamine receptor antagonists likely to enhance HDAC5 phosphorylation through inhibition of PP2A

**Fig 3:**
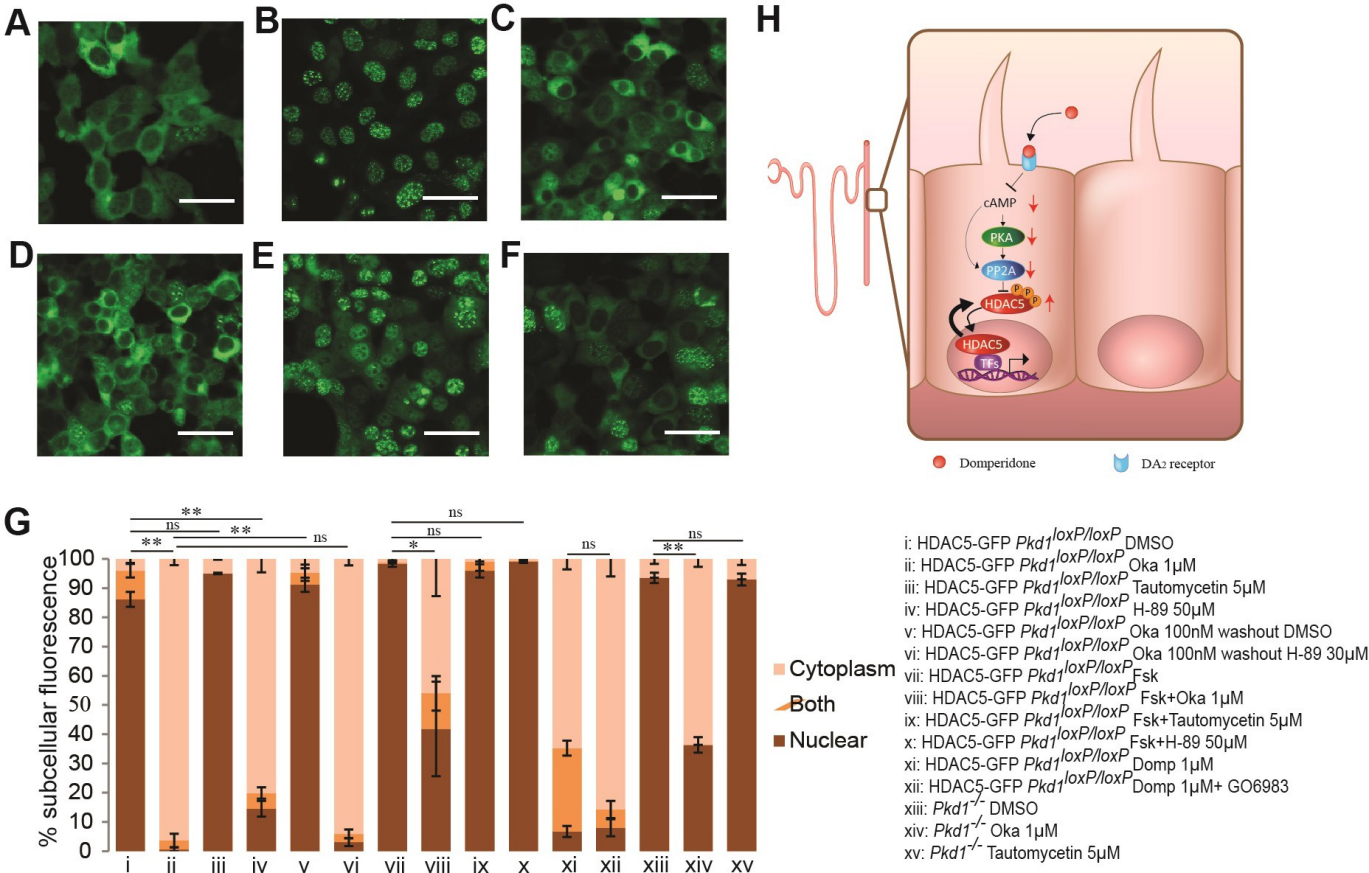
PP2A inhibition downstream of PKA activation is crucial for nuclear export of HDAC5. To investigate if direct or indirect inhibition of the phosphatase PP2A modulates the HDAC5 nuclear-cytoplasmic shuttling, HDAC5-GFP-expressing *PKD1*^*loxP/loxP*^ cells were treated with drugs that are known to inhibit PP2A or its regulating kinase, PKA. (A) Representative image shows that 1 µM Oka, that can selectively inhibit PP2A, can cause robust export of HDAC5. (B) However inhibition of PP1, using a selective inhibitor tautomycetin at 5 μM cannot cause HDAC5 export. (C) PKA inhibition using a potent inhibitor H-89 (50 μM) can cause HDAC5 export. (D,E) After treating the cells with 100 nM Oka, the drug was washed out using media containing 30 μM H-89 (D) or DMSO (E). Presence of H-89 in the washout media, prevented HDAC5 from returning back to the nucleus, suggesting a role for PKA in the nuclear import of HDAC5. (F) Oka can also cause moderate export of HDAC5 in cells treated with Fsk that have elevated cAMP level. (G) Quantification of nuclear export of HDAC5 following various treatments are plotted. PKC inhibition by GÖ6983 could not inhibit the export caused by domperidone. Domperidone data is same as in Fig 1A. Each bar is an average of >3 independent experiments (> 100 cells per experiment). Error bars: SEM. Scale bars: 40 μm. *, P value < 0.05 and **, P value <1x 10^−4^ for nuclear population. (H) Working model showing how domperidone regulates HDAC5 nuclear export in the collecting duct cells of the nephron. Domperidone binding can somehow inhibit PP2A, directly or indirectly via PKA. This can increase HDAC5 phosphorylation and thus cause nuclear export of HDAC5 and de-repression of target genes. Red arrows depict the functional consequence of domperidone binding to the DA_2_ receptor.

We next investigated the mechanism by which dopamine receptor antagonists stimulate nuclear import or export of HDAC5. It has been well-established that HDAC5 nuclear export is caused by phosphorylation of serine residues near its NLS, leading to 14-3-3 binding. We reasoned that reduced PP2A activity or increased PKA activity can result in hyper-phosphorylation and nuclear export of HDAC5. Supporting this hypothesis, inhibition of PP2A by using okadaic acid (Oka) at a concentration selective for PP2A [45, 46] promoted nuclear export of HDAC5 (Fig 3A and 3G). By contrast, HDAC5 was not exported from the nucleus when cells were treated with tautomycetin, a selective inhibitor for protein phosphatase 1 [47] (Fig 3B and 3G). Previous evidence suggests that PP2A may be activated by cAMP directly [48] or indirectly via protein kinase A [49, 50]. We therefore tested the effect of inhibition of PKA by using a potent competitive inhibitor, H-89. H-89 induced robust export of HDAC5 from the nucleus (Fig 3C and 3G). Because Oka is a reversible inhibitor, we also tested if HDAC5 returns to the nucleus after washout of the drug, and if so, whether this process is dependent on PKA. Indeed, HDAC5 export was reversed after Oka washout and this process was inhibited by H-89 (Fig 3D, 3E and 3G). Oka, but not tautomycetin, also caused robust HDAC5 export in the presence of forskolin (Fsk) (Fig 3F and 3G), further establishing the epistatic relationship between PP2A and protein kinase A activation. Our previous study found that HDAC5 phosphorylation and export in response to fluid flow was inhibited by GÖ6983, a broad spectrum inhibitor of protein kinase C (PKC) [12]. However, GÖ6983 could not inhibit the HDAC5 export caused by domperidone (Fig 3G). These data suggest that dopamine receptor antagonist enhances HDAC5 phosphorylation and nuclear export through inhibition of PP2A rather than stimulation of the upstream kinase (Fig 3H).

### Domperidone reduces cyst formation in *Pkd1*^*–/–*^mice

**Fig 4:**
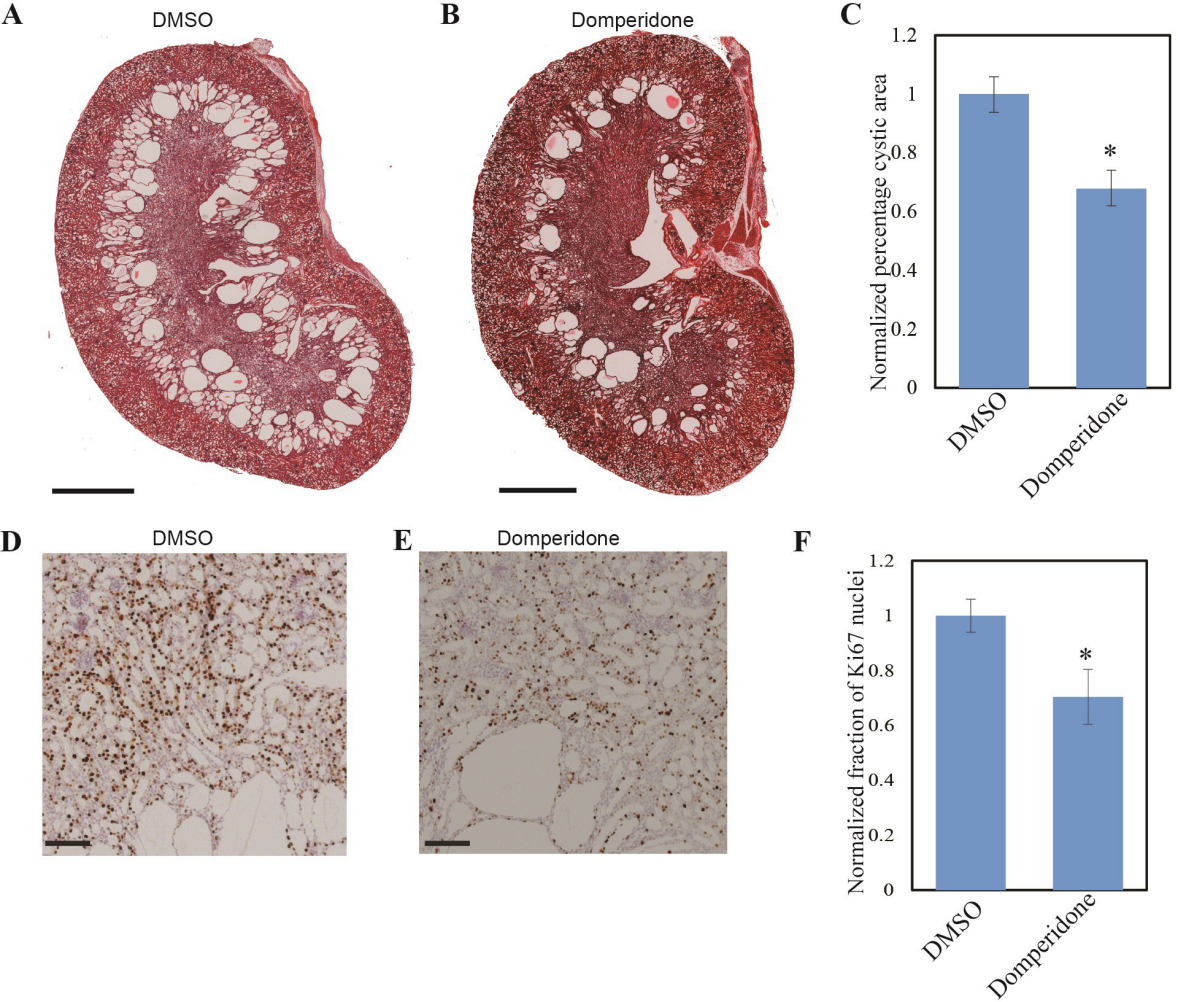
Domperidone treatment reduces cystic growth in *Pkd1*^*–/–*^mice. Tamoxifen treated *Pkd1*^*loxP/loxP*^ *EsrCre* mice were injected with 10 mg/kg domperidone or same volume of DMSO from P12-P20 and the kidneys were dissected on P21. (A, B) Representative H&E stained kidney sections of domperidone treated (B) or vehicle treated (A) mice from the same litter show a reduction in cystic area in domperidone treated mice. (C) Percentage of cystic area over total kidney section area normalized to control group is shown in the bar plot. Shown are mean ± SEM of all sections quantified for each group. *, P<0.005 compared with control. n = 9 for DMSO or domperidone-treated group (both kidneys were used in quantification for each animal). (D-F) Representative Ki67 staining of vehicle-treated (D), or domperidone-treated (E) kidney sections, show decreased cell proliferation in the treated kidney sections. (F) Normalized fraction of Ki67 positive nuclei over total number of nuclei shows a significant decrease in Ki67 positive nuclei in domperidone treated mice. Scale bars: 1mm (A and B), 100 μm (D and E). *, P<0.005 compared with control. n = 9 for DMSO or domperidone-treated group.

**Fig 5:**
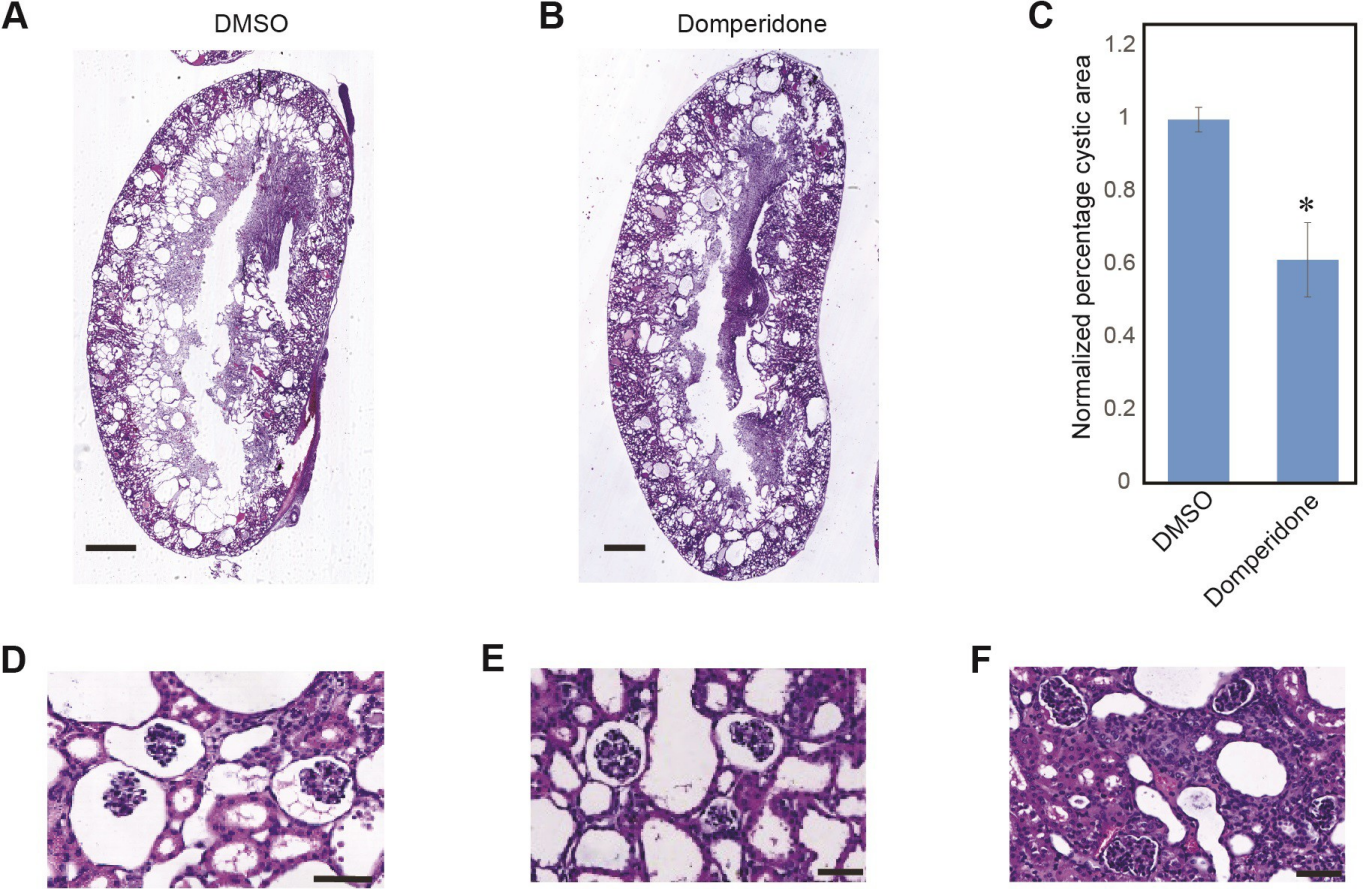
Domperidone further reduce cystic area in *Pkd1*^*–/–*^mice following longterm treatment. Tamoxifen treated *Pkd1*^*loxP/loxP*^ *EsrCre* mice were injected with 10 mg/kg domperidone or same volume of DMSO from P12-P42 and the kidneys were dissected on P43. (A, B) Representative H&E stained kidney sections of domperidone treated (B) or vehicle treated (A) mice from the same litter show a reduction in cystic area in domperidone treated mice. (C) Percentage of cystic area over total kidney section area normalized to control group is shown in the bar plot. Shown are mean ± SEM of all sections quantified for each group. *, P<0.005 compared with control. n = 2 mice for DMSO and n = 3 mice for domperidone-treated group (both kidneys were used in quantification for each animal). (D) Representative kidney sections showing dilation of Bowman’s capsules in vehicle-treated, which is reduced in domperidone-treated mice (E, F). Scale bars: 1mm (A and B), 50 μm (D, E and F).

Combining the findings that genetic inhibition of HDAC5 can suppress cyst formation and dopamine antagonists promotes robust HDAC5 nuclear export, we hypothesized that the dopamine antagonist can impede cyst development in ADPKD mice. We injected *Pkd1*^*–/–*^mice (gene disruption induced by EsrCre activation at P10-P11) intraperitoneally with domperidone or vehicle control from P12 to P20 (see Methods). Kidneys were harvested at P21 and the cystic areas were quantified. Domperidone treatment significantly reduced the percentage of cystic area compared to vehicle treatment (Fig 4A–4C; due to the fact that different litters exhibited different extent of cyst development, normalized cystic area percentages are presented). This reduction in the cystic area was accompanied by decreased cell proliferation, as indicated by Ki67 staining (Fig 4D–4F). We further treated few mice for longer time, from P12-P42 and harvested the kidneys at P43 (see methods). Domperidone treatment further reduced cyst progression with reduction in cortical cyst (Fig 5A–5C) and increased body weight compared to DMSO treated mice (S3 Table). In addition, there was marked reduction in the glomerular cysts in domperidone treated mice (Fig 5D–5F).

## Discussion

Class IIa HDACs confer transcriptional repression through recruitment of co-repressors and other histone-modifying enzymes such as Class I HDACs [51–53]. The interaction between class IIa HDACs and MEF2 family transcription factors is a common target of mechanosensory pathways in cardiac myocytes [53], blood vessels [54] and renal tubule cells [12]. The results described in this study in combination with previous work demonstrate that HDAC5, a class IIa HDAC, is a target of polycystin-mediated fluid sheer stress sensation and plays a role in cyst formation and the development of ADPKD. Genetic inhibition of HDAC5, even heterozygosity, can suppress cyst formation caused by the *Pkd1* mutation, providing the genetic basis supporting HDAC5 as a therapeutic target for ADPKD disease intervention. However, because HDAC5, like other class IIa HDAC members, lacks intrinsic histone deacetylase activity, the conventional approach of screening for enzymatic inhibitors is unlikely to be fruitful. Our screen design took advantage of the knowledge that the nucleo-cytoplasmic shuttling of HDAC5 regulates its activity as a transcriptional repressor [17, 55, 56] and was aimed at targeting the signaling network that regulates HDAC5. Unexpectedly, our screen revealed that dopamine antagonists cause the nuclear export of HDAC5 in a PKD1- and cilia-independent manner. Loxapine succinate is a FDA approved antipsychotic drug. Domperidone was previously approved by FDA but was recently removed from the market in the US because of its potential harmful effect on children when it is used for increasing lactation by increasing prolactin levels and its association with risk of cardiac arrhythmia in older patients. Nevertheless, domperidone is a well-accepted drug for treating severe nausea and gastric reflux and is widely used in Canada and Europe [57].

Our previous work discovered that calcium signaling in response to fluid-flow induces HDAC5 export from the nucleus and that this is impaired in *Pkd1*^*–/–*^mouse kidney cells [12]. The HDAC5-nuclear export pathway is strikingly reminiscent of that mediating cardiac hypertrophy in response to increasing cardiovascular stress, where HDAC nuclear export enables activation of MEF2 target genes required for hypertrophic growth of cardiac musculature [32, 55, 58, 59]. This led us to hypothesize that the renal HDAC5 regulation in response to increased fluid stress may also play a role in strengthening epithelial function and architecture and, thus, preventing renal injury and cyst development. Our small molecule screen in this study unexpectedly found a class of GPCR antagonists that have the ability to rescue this potential renal protection pathway in *Pkd1* mutant cells. Furthermore, the rescuing mechanism appears to occur via a pathway that boosts HDAC5 phosphorylation working in parallel to that downstream of polycystin activity: while polycystins promote kinase activity toward HDAC5, dopamine antagonists act to inhibit the phosphatase activity against HDAC5. Our finding thus provides an interesting example where the outcome of a biological pathway is rescued as a result of the complexity and redundancy that is commonly present in regulatory networks.

Apart from the renal abnormality, ADPKD is characterized by vascular hypertension. Impaired ciliary function in PKD leads to impaired nitric oxide biosynthesis and was suggested as one of the reasons for vascular hypertension [60, 61]. Dopamine has been shown to increase cilia length in endothelial cells [39] and epithelial cells [40] via DR5, a DA_1_ family member. Thus dopamine and DA_1_ agonists have been suggested as therapy for hypertension in PKD patients. DA_1_ receptors are localized to the smooth muscle of renal arteries, juxtaglomerular apparatus and renal tubules, with higher density in the proximal tubule [44, 62]. Activation of DA_1_ is responsible for the natriuretic effect of dopamine in the kidney. Further, disruption of DA_1A_ receptors has been shown to cause hypertension in mice [63]. DA_2_ receptors are expressed in the intimal layer of renal vasculature, glomerulus, sympathetic nerve terminal and renal tubules [62, 64], but their function in the kidney is less understood. We show in this study that specific inhibition of DA_2_ receptors, by domperidone, loxapine succinate, spiperone, and haloperidol, can cause the nuclear export of HDAC5 independent of cilia or polycystine-1. Further research is required to elucidate the mechanism underlying the drug action. Nevertheless, the anti-proliferative effect and reduction of renal cyst growth by domperidone in ADPKD mice make this class of drugs promising candidates to be explored for therapy in ADPKD patients. Since nucleocytoplasmic shuttling of class-IIa HDACs is implicated in several other diseases or conditions, our approach may have broad therapeutic potential [20, 65, 66].

## Supporting information

S1 Fig Genetic studies show that inhibition of *Hdac5* can be beneficial in ADPKD mice. *Pkd1*^*loxP/loxP*^ *Hdac5* ^*+/–*^mice were crossed to obtain *Pkd1*^*–/–*^*Hdac5*^*+/+*^, *Pkd1*^*–/–*^*Hdac5*^*+/–*^mice and then their kidneys were dissected at P21 and P28. Representative H&E stained histology sections of kidneys from P21 *Pkd1*^*–/–*^*Hdac5*^*+/+*^ (A) and P21 *Pkd1*^*–/–*^*Hdac5*^*+/–*^(B) from littermates show a reduction in cystic area in *Hdac5* heterozygote mice compared to *Hdac5* homozygote mice. (C) Normalized percentage of cystic areas over total kidney section areas of different genotypes are shown, as indicated. Shown are mean ± SEM of all sections quantified for each genotype (For P21, n = 5 for *Pkd1*^*–/–*^*Hdac5*^*+/+*^ and n = 11 for *Pkd1*^*–/–*^*Hdac5*^*+/–*^. For P28, n = 2 for both *Pkd1*^*–/–*^*Hdac5*^*+/+*^ and *Pkd1*^*–/–*^*Hdac5*^*+/–*^). *, P<0.005 compared with *Pkd1*^*–/–*^*Hdac5*^*+/+*^. (D) Quantification of blood urea nitrogen in mg/dl ± SEM for different genotypes at P21and P28 (n = 3 for P21 *Pkd1*^*–/–*^*Hdac5*^*+/+*^, n = 11 for P21 *Pkd1*^*–/–*^*Hdac5*^*+/–*^, n = 4 for P28 *Pkd1*^*–/–*^*Hdac5*^*+/+*^ and n = 4 for P28 *Pkd1*^*–/–*^*Hdac5*^*+/–*^). All the BUN values have been normalized to the *Pkd1*^*–/–*^*Hdac5*^*+/+*^ value at P21. *, P<0.05 compared to age matched *Pkd1*^*–/–*^*Hdac5*^*+/+*^.
(TIF)

S2 Fig Small-molecule screen identifies receptor antagonists as positive hits and receptor agonists as negative hits. (A) Schematic representation of the small-molecule screen pipeline is shown. (B) Chemical structure of the two dopamine antagonists, domperidone and loxapine succinate. From the hit list, we selected compounds that are known GPCR ligands. The majority of the positive hits are receptor antagonists (C), while most of the negative hits are receptor agonists (D). Antagonists are depicted by green bars and agonists are depicted by grey bars. Negative control (DMSO) and positive controls (PMA and I3A) are shown in each graph. Shown are mean ± SEM. The dashed line depicts the cutoff value of three standard deviation difference from DMSO. Bars with letters ‘fp’ and ‘v’ depict the ones that were validated manually. ‘fp’ depict the ones that did not validate (false positive) and ‘v’ depict the ones that validated.
(TIF)

S3 Fig Terbutaline promotes import of flow or PMA-exported HDAC5. HDAC5-GFP-expressing *PKD1*^*loxP/loxP*^ cells were either subjected to fluid-flow using media containing DMSO or terbutaline hemisulfate or treated with PMA in presence of DMSO or terbutaline hemisulfate. (A, C) In the presence of terbutaline hemisulfate, HDAC5-GFP localizes primarily in the nucleus suggesting that both the flow and PMA fails to cause HDAC5 export in its presence or it returns back to the nucleus following the export. Error bar: Mean ± SEM of >3 independent experiment. (B, C) Domperidone caused export of HDAC5-GFP in *PKD1*^*loxP/loxP*^ cells transiently transfected with HDAC5-GFP. *, P value < 0.005 and **, P value <1x10^-6^ for nuclear population.
(TIF)

S4 Fig *Kif3a*^*–/–*^cells are devoid of primary cilia. In order to confirm that *Kif3a*^*–/–*^cells are devoid of cilia these cells were stained with acetylated tubulin that decorate the cilia. Representative max projected images show that cilia is absent in *Kif3a*^*–/–*^cells (B) compared to wild-type cells as can be seen from acetylated tubulin staining (red, *). The nuclei are stained with DAPI (blue). The bottom panel shows the orthogonal view of the cilia which is present in wild-type (wt) but not in *Kif3a*^*–/–*^cells. (C) *Kif3a*^*–/–*^cells were subjected to treatment with DMSO, 1 μM domperidone or 1 μM loxapine succinate, as indicated. Scale bars: 20 μm. (D) Quantification of percentage of cells with HDAC5-GFP in the cytoplasm, nucleus, or both compartments. Bar graphs show averages from > 3 independent experiments. Error bars: standard error of the mean (SEM). * indicate P value < 1x 10^−5^ for nuclear population compared to the respective DMSO control. For raclopride, nuclear population is compared to DMSO control in Fig 1C.
(TIF)

S5 Fig DBA staining in *Pkd1*^*loxP/loxP*^ cells. *Pkd1*^*loxP/loxP*^ cells were stained with DBA (green) and DAPI (blue).
(TIF)

S1 Table Positive hit compounds. Mechanism of action listed are taken from the Prestwick library annotation except the ones in green, which are based on literature. Also fraction nuclear, SEM and P values are tabulated (for details see Methods).
(PDF)

S2 Table Negative hit compounds. Mechanism of action listed are taken from the Prestwick library annotation except the ones in green, which are based on literature.
(PDF)

S3 Table Effect of domperidone treatment on body weight (BW), kidney weight (KW) and number of glomerular cyst in long term treatment group. Measurements made in P43 mice.
(PDF)

S1 Checklist NC3Rs arrive guidelines checklist-parama. (PDF)

S1 File Fig 1 Uncropped blot. (PDF)

S2 File Fig 2 Uncropped blot. (PDF)

S1 Data Raw data. (PDF)
