# Supplementary figures and images for: Dopamine receptor antagonists as potential therapeutic agents for ADPKD
Source: PLoS One. 2019 May 6;14(5):e0216220. doi: 10.1371/journal.pone.0216220 (PMC6502331; doi:10.1371/journal.pone.0216220)

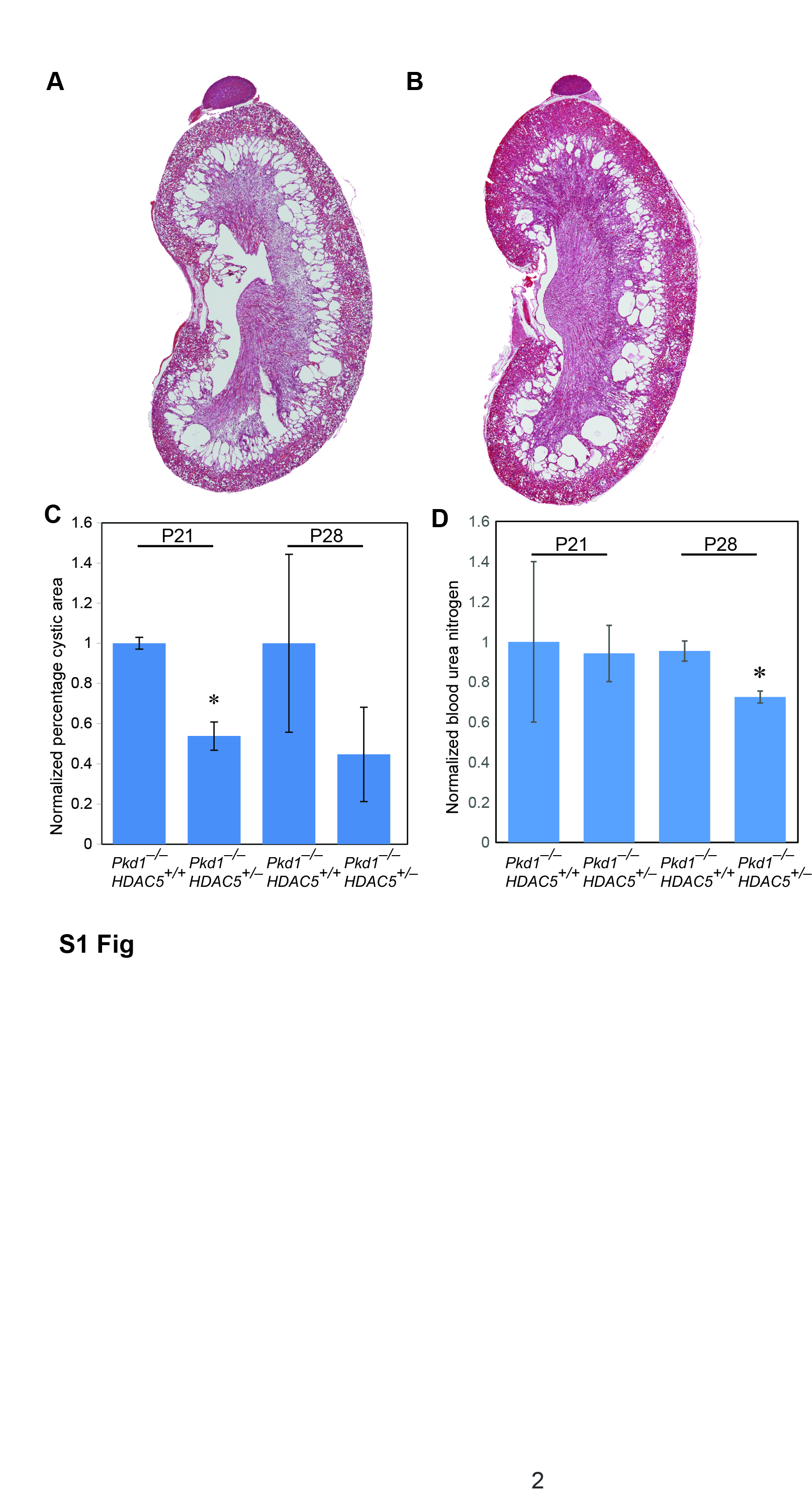

Supplement: S1 Fig — Pkd1loxP/loxP Hdac5 +/–mice were crossed to obtain Pkd1–/–Hdac5+/+, Pkd1–/–Hdac5+/–mice and then their kidneys were dissected at P21 and P28. Representative H&E stained histology sections of kidneys from P21 Pkd1–/–Hdac5+/+ (A) and P21 Pkd1–/–Hdac5+/–(B) from littermates show a reduction in cystic area in Hdac5 heterozygote mice compared to Hdac5 homozygote mice. (C) Normalized percentage of cystic areas over total kidney section areas of different genotypes are shown, as indicated. Shown are mean ± SEM of all sections quantified for each genotype (For P21, n = 5 for Pkd1–/–Hdac5+/+ and n = 11 for Pkd1–/–Hdac5+/–. For P28, n = 2 for both Pkd1–/–Hdac5+/+ and Pkd1–/–Hdac5+/–). *, P<0.005 compared with Pkd1–/–Hdac5+/+. (D) Quantification of blood urea nitrogen in mg/dl ± SEM for different genotypes at P21and P28 (n = 3 for P21 Pkd1–/–Hdac5+/+, n = 11 for P21 Pkd1–/–Hdac5+/–, n = 4 for P28 Pkd1–/–Hdac5+/+ and n = 4 for P28 Pkd1–/–Hdac5+/–). All the BUN values have been normalized to the Pkd1–/–Hdac5+/+ value at P21. *, P<0.05 compared to age matched Pkd1–/–Hdac5+/+. (TIF) [file pone.0216220.s001.tif]

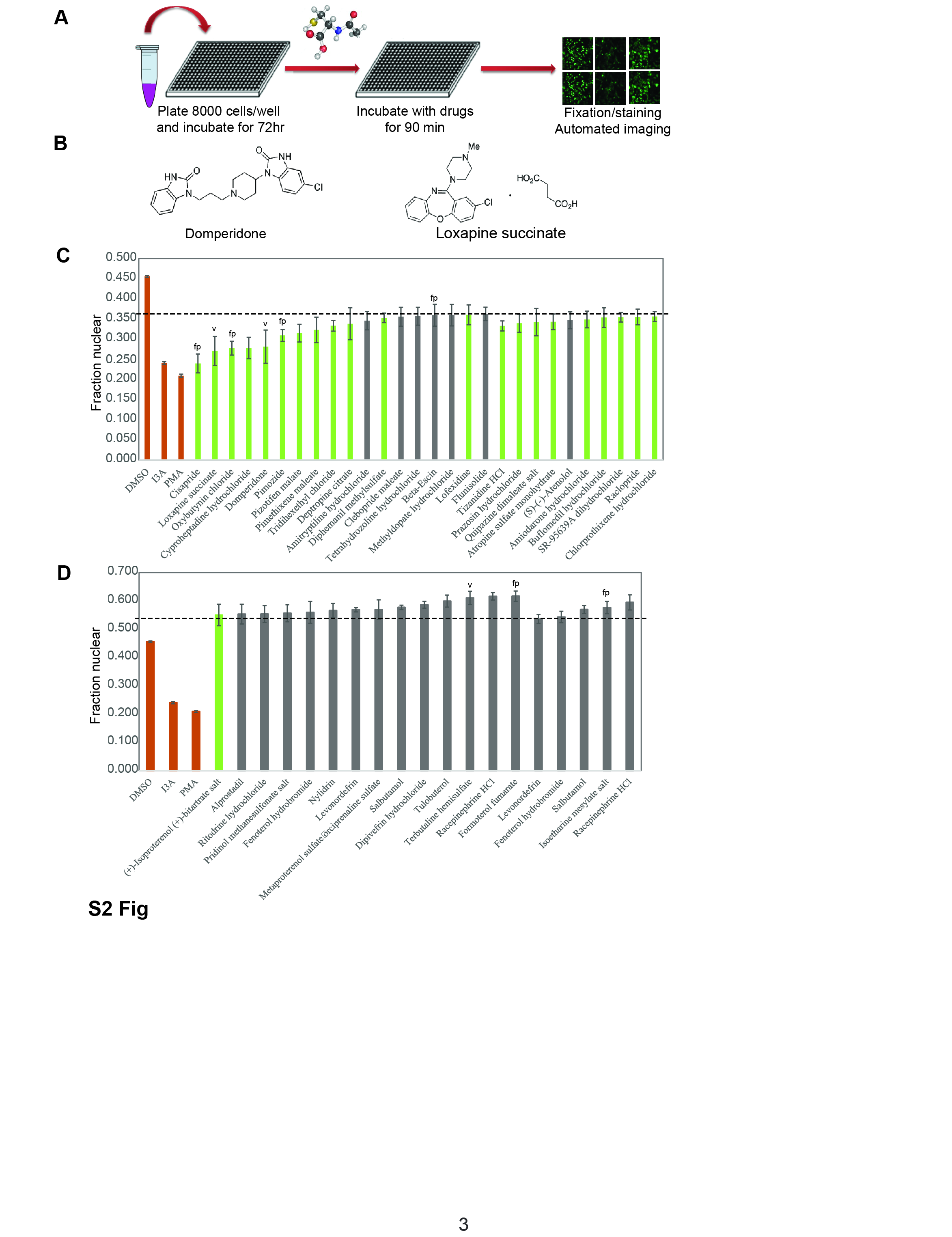

Supplement: S2 Fig — (A) Schematic representation of the small-molecule screen pipeline is shown. (B) Chemical structure of the two dopamine antagonists, domperidone and loxapine succinate. From the hit list, we selected compounds that are known GPCR ligands. The majority of the positive hits are receptor antagonists (C), while most of the negative hits are receptor agonists (D). Antagonists are depicted by green bars and agonists are depicted by grey bars. Negative control (DMSO) and positive controls (PMA and I3A) are shown in each graph. Shown are mean ± SEM. The dashed line depicts the cutoff value of three standard deviation difference from DMSO. Bars with letters ‘fp’ and ‘v’ depict the ones that were validated manually. ‘fp’ depict the ones that did not validate (false positive) and ‘v’ depict the ones that validated. (TIF) [file pone.0216220.s002.tif]

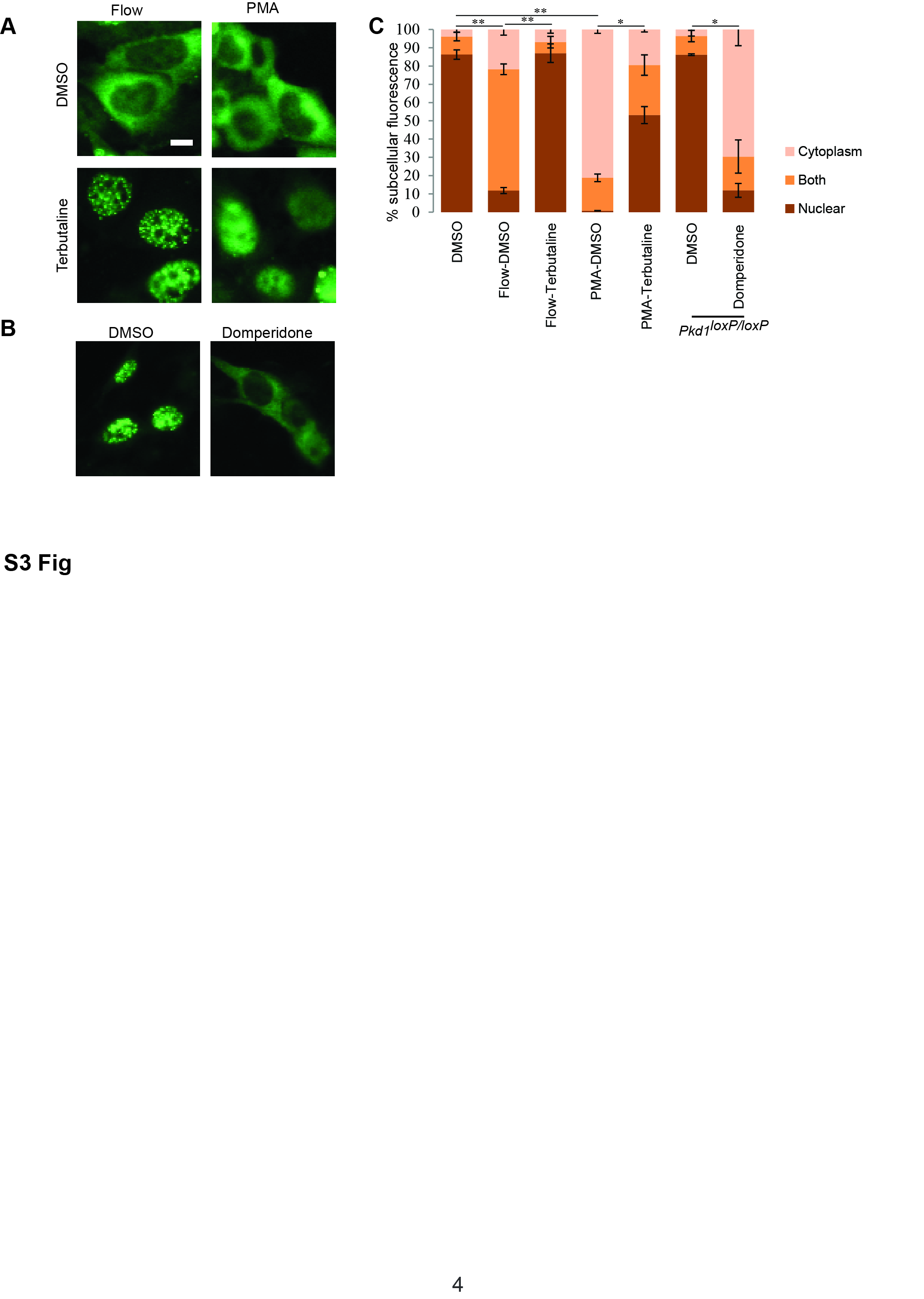

Supplement: S3 Fig — HDAC5-GFP-expressing PKD1loxP/loxP cells were either subjected to fluid-flow using media containing DMSO or terbutaline hemisulfate or treated with PMA in presence of DMSO or terbutaline hemisulfate. (A, C) In the presence of terbutaline hemisulfate, HDAC5-GFP localizes primarily in the nucleus suggesting that both the flow and PMA fails to cause HDAC5 export in its presence or it returns back to the nucleus following the export. Error bar: Mean ± SEM of >3 independent experiment. (B, C) Domperidone caused export of HDAC5-GFP in PKD1loxP/loxP cells transiently transfected with HDAC5-GFP. *, P value < 0.005 and **, P value <1x10^-6^ for nuclear population. (TIF) [file pone.0216220.s003.tif]

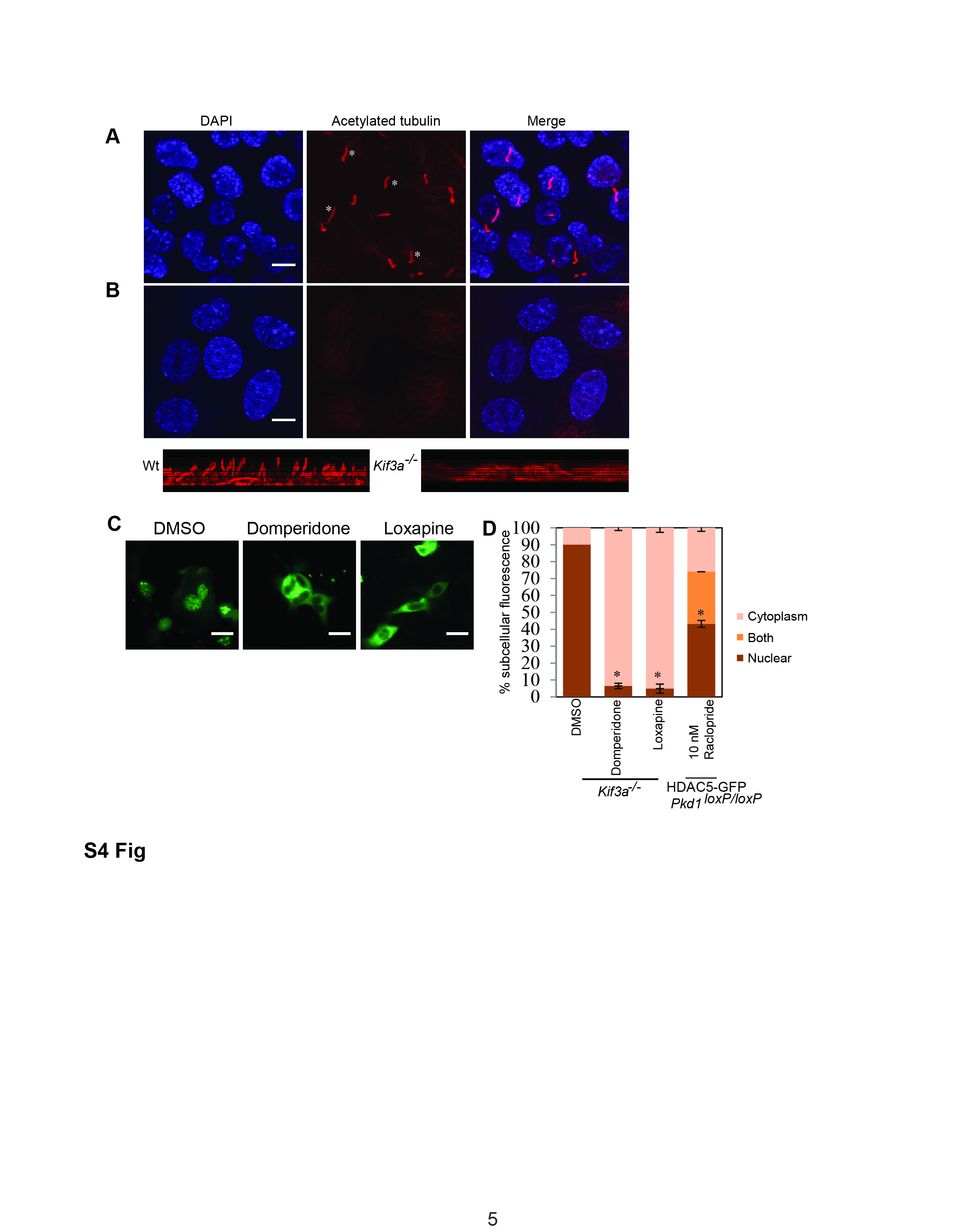

Supplement: S4 Fig — In order to confirm that Kif3a–/–cells are devoid of cilia these cells were stained with acetylated tubulin that decorate the cilia. Representative max projected images show that cilia is absent in Kif3a–/–cells (B) compared to wild-type cells as can be seen from acetylated tubulin staining (red, *). The nuclei are stained with DAPI (blue). The bottom panel shows the orthogonal view of the cilia which is present in wild-type (wt) but not in Kif3a–/–cells. (C) Kif3a–/–cells were subjected to treatment with DMSO, 1 μM domperidone or 1 μM loxapine succinate, as indicated. Scale bars: 20 μm. (D) Quantification of percentage of cells with HDAC5-GFP in the cytoplasm, nucleus, or both compartments. Bar graphs show averages from > 3 independent experiments. Error bars: standard error of the mean (SEM). * indicate P value < 1x 10^−5^ for nuclear population compared to the respective DMSO control. For raclopride, nuclear population is compared to DMSO control in Fig 1C. (TIF) [file pone.0216220.s004.tif]

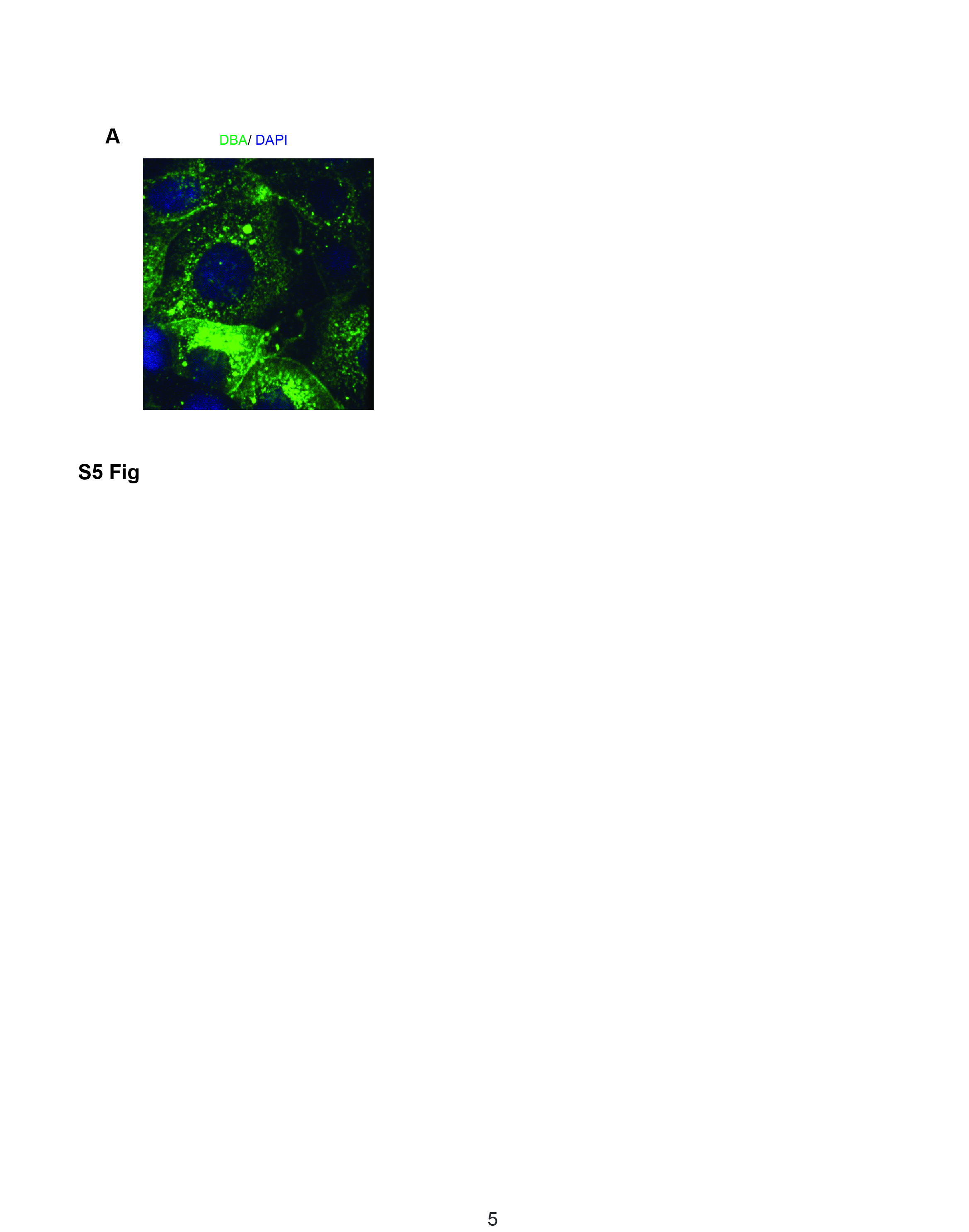

Supplement: S5 Fig — Pkd1loxP/loxP cells were stained with DBA (green) and DAPI (blue). (TIF) [file pone.0216220.s005.tif]

Full unedited gel for Fig 1

DMSO

Loxapine

Domperidone

S100

NE

S100

NE

S100

NE

HDAC5-P

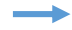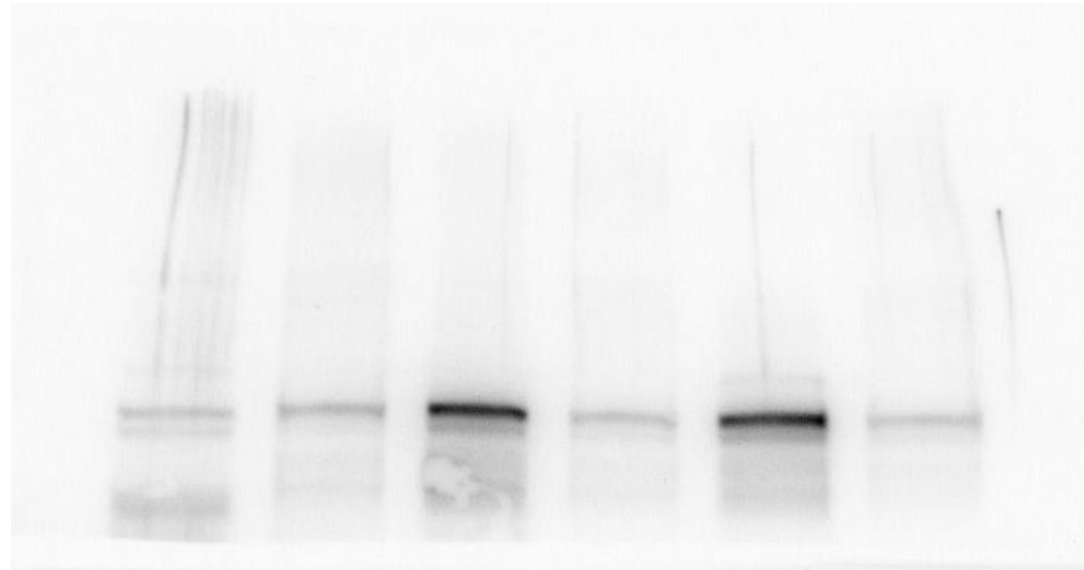

Lamin B1

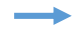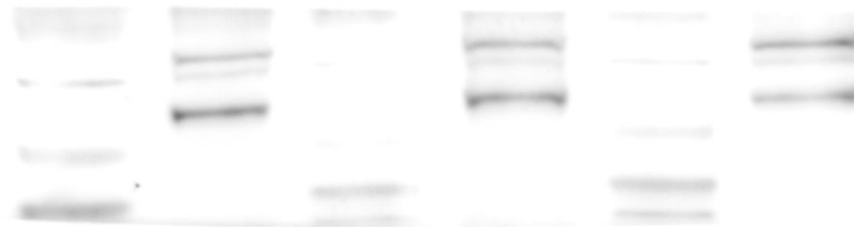

14-3-3

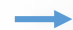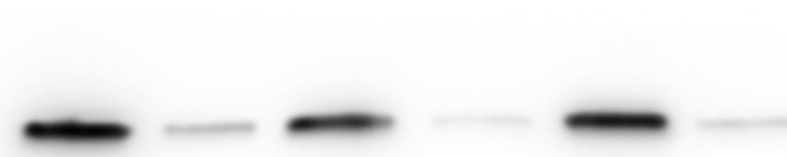

Supplement: S1 File — (PDF) [file pone.0216220.s010.pdf]
