# Supplementary material for: Dopamine receptor antagonists as potential therapeutic agents for ADPKD
Source: PLoS One. 2019 May 6;14(5):e0216220. doi: 10.1371/journal.pone.0216220 (PMC6502331; doi:10.1371/journal.pone.0216220)
Supplement: S1 Table — Mechanism of action listed are taken from the Prestwick library annotation except the ones in green, which are based on literature. Also fraction nuclear, SEM and P values are tabulated (for details see Methods). (PDF) [file pone.0216220.s006.pdf]

S1 Table. Positive hit compounds. Mechanism of action listed are taken from the Prestwick library annotation except the ones in green, which are based on literature. Also fraction nuclear, SEM and P values are tabulated (for details see Methods).

	Chemical name	Mechanism of action	Fraction Nuclear	SEM	P
1uM positive hits	Cisapride	5-HT antagonist	0.240	0.023	0.0002
	Loxapine succinate	Dopamine antagonist	0.270	0.036	0.0036
	Oxybutynin chloride	Anticholinergic	0.277	0.017	0.0001
	Cyproheptadine hydrochloride	5-HT antagonist; 'Histamine antagonist	0.278	0.026	0.0011
	Domperidone	Dopamine Antagonists	0.281	0.041	0.0083
	Pimozide	Dopamine antagonist ?	0.309	0.015	0.0002
	Pizotifen malate	Serotonin antagonist	0.315	0.022	0.0013
	Pimethixene maleate	Anticholinergic, Serotonin antagonist, Bradykinin antagonist	0.323	0.032	0.0084
	Tridihexethyl chloride	Anticholinergic	0.333	0.013	0.0003
	Deptropine citrate	Antihistamine, anticholinergic	0.338	0.040	0.0313
	Amitryptiline hydrochloride	Alpha 1 antagonist, anticholinergic, histaminic antagonist, serotonine uptake inhibitor, noradrenaline	0.346	0.023	0.0048
	Nisoldipine	Calcium channel blocker	0.347	0.012	0.0002
	Folic acid	Member of Vitamin B family	0.348	0.021	0.0035
	Cefamandole sodium salt	Bacterial transpeptidase inhibitor	0.349	0.013	0.0005
	Carbadox	Antibiotic	0.350	0.011	0.0002
	Atovaquone	Inhibits parasitic electron transport chain	0.351	0.005	0.0000
	Brinzolamide	Carbonic anhydrase inhibitor	0.352	0.026	0.0099
	Diphemanil methylsulfate	Anticholinergic	0.353	0.012	0.0003
	Etanidazole	DNA damage	0.353	0.017	0.0016
	Hycanthone	DNA intercaling agent	0.354	0.016	0.0014
	Bacampicillin hydrochloride	Bacterial transpeptidase inhibitor	0.355	0.012	0.0004
	Clebopride maleate	Dopamine antagonist, stimulate the intramural cholinergic system	0.355	0.024	0.0080
	Erlotinib	Tyrosine kinase inhibitor	0.356	0.019	0.0035
	Tetrahydrozoline hydrochloride	Adrenergic agonist	0.357	0.022	0.0066
	Acemetacin	Cyclooxygenase inhibitor	0.358	0.024	0.0087
	Propylthiouracil	Antimetabolite	0.359	0.027	0.0150
	Beta-Escin	Induce nitric oxide synthesis, serotonin antagonist, histamine antagonist	0.359	0.027	0.0160
	Methyldopate hydrochloride	Adrenergic receptor agonist	0.359	0.027	0.0153
	Lofexidine	alpha2-adrenergic receptor agonist	0.360	0.025	0.0116
	Fluoxetine hydrochloride	5-HT uptake inhibitor	0.360	0.012	0.0006

	Fluspirilen	Probable weak calcium channel antagonist	0.361	0.027	0.0179
	Ceforanide	Antibiotic	0.361	0.021	0.0061
	Flunisolide	Glucocorticoid receptor agonist, anti-inflammatory	0.363	0.016	0.0024
100nM positive hits	Etofenamate	cyclooxygenase inhibitor	0.319	0.026	0.0048
	Doxorubicin hydrochloride	DNA intercalant	0.329	0.027	0.0079
	Trioxsalen	Photosenitization causing programmed cell death	0.331	0.018	0.0013
	Tiabendazole	Microtubule inhibitor	0.332	0.018	0.0015
	Tizanidine HCl	Noradrenergic receptor agonist	0.333	0.012	0.0003
	Mercaptopurine	inhibits purine nucleotide synthesis and metabolism	0.337	0.031	0.0167
	Probenecid	Uric acid uptake inhibitor	0.337	0.015	0.0008
	Florfenicol	Protein synthesis inhibitor	0.338	0.031	0.0174
	Prazosin hydrochloride	Adrenergic receptor antagonist	0.339	0.022	0.0051
	Stavudine	nucleoside analog reverse transcriptase inhibitor	0.340	0.010	0.0001
	Abacavir Sulfate	Nucleoside analog reverse transcriptase inhibitor	0.342	0.026	0.0109
	Quipazine dimaleate salt	5-HT agonist; '5-HT3 ligand	0.342	0.034	0.0282
	Tolazamide	ATP-sensitive K+ ion channels blocker	0.342	0.047	0.0779
	Imipenem	Bacterial transpeptidase inhibitor	0.343	0.019	0.0031
	Atropine sulfate monohydrate	Muscarinic antagonist	0.343	0.020	0.0032
	Carbimazole	Iodine oxidazing inhibitor	0.343	0.011	0.0003
	Colistin sulfate	Performs membrane ionophores	0.345	0.012	0.0004
	Acarbose	inhibits enzymes (glycoside hydrolases) needed to digest carbohydrates	0.345	0.024	0.0077
	Meclocycline sulfosalicylate	Ribosomal protein synthesis inhibitor	0.346	0.025	0.0101
	(S)-(-)-Atenolol	Adrenergic receptor antagonist	0.347	0.021	0.0052
	Rimantadine Hydrochloride	Antiviral	0.347	0.032	0.0262
	Carbadox	Antibacterial	0.347	0.029	0.0194
	Valacyclovir hydrochloride	inhibitor of viral DNA polymerase	0.348	0.025	0.0113
	Tomoxetine hydrochloride	norepinephrine reuptake inhibitor	0.348	0.014	0.0010
	Amiodarone hydrochloride	Na+ channel blocker; 'K+ channel blocker; 'Non-competitive beta-adrenergic blocker	0.349	0.021	0.0053
	Prenylamine lactate	Calcium channel activator	0.350	0.006	0.0000
	Probucol	Anti-hyperlipidemic	0.351	0.027	0.0160
	Proadifen hydrochloride	Cytochrome P450 mono-oxygenases inhibitor; 'Na+ channel blocker	0.352	0.023	0.0100
	Buflomedil hydrochloride	Vasodilation, adrenoceptor antagonist	0.353	0.024	0.0124
	Omeprazole	Non competitive ATPase H+ pump inhibitor	0.354	0.019	0.0045
	Ethaverine hydrochloride	Improve cochlear microcirculation	0.354	0.020	0.0055

	SR-95639A dihydrochloride	M1 agonist receptor	0.354	0.012	0.0006
	Raclopride	selective dopamine D1/D2 receptors antagonist	0.355	0.019	0.0053
	Pentobarbital	Inhibition of calcium channel	0.355	0.026	0.0175
	Canrenoic acid potassium salt	Detergent	0.356	0.013	0.0008
	Chlorprothixene hydrochloride	D2 dopamine receptor antagonist, GABAA receptors antagonist	0.356	0.012	0.0007
	Tiaprofenic acid	Cyclooxygenase inhibitor	0.356	0.022	0.0091
