# Supplementary material for: Dopamine receptor antagonists as potential therapeutic agents for ADPKD
Source: PLoS One. 2019 May 6;14(5):e0216220. doi: 10.1371/journal.pone.0216220 (PMC6502331; doi:10.1371/journal.pone.0216220)
Supplement: S2 Table — Mechanism of action listed are taken from the Prestwick library annotation except the ones in green, which are based on literature. (PDF) [file pone.0216220.s007.pdf]

**S2 Table. Negative hit compounds**. Mechanism of action listed are taken from the Prestwick library annotation except the ones in green, which are based on literature.

| | Chemical name | Mechanism of action | Fraction Nuclear | SEM | P |
|---|---|---|---|---|---|
| 1uM negative hits | (+)-Isoproterenol (+)-bitartrate salt | Adernergic receptor agonist | 0.550 | 0.038 | 0.0547 |
| | Alprostadil | Prostaglandin receptor agonist | 0.553 | 0.035 | 0.0386 |
| | Ritodrine hydrochloride | Beta2 agonist | 0.553 | 0.029 | 0.0209 |
| | Pridinol methanesulfonate salt | Anticholinergic | 0.556 | 0.030 | 0.0205 |
| | Fenoterol hydrobromide | Beta adrenergic agonist | 0.559 | 0.039 | 0.0446 |
| | Benzethonium chloride | Detergent | 0.562 | 0.037 | 0.0350 |
| | Acetohexamide | Blocking of ATP-sensitive K+ channel; Stimulation of insulin release | 0.564 | 0.021 | 0.0037 |
| | Nylidrin | sympathomimetic beta-adrenergic agonist | 0.566 | 0.026 | 0.0077 |
| | Levonordefrin | Adrenergic receptor agonist | 0.569 | 0.008 | 0.0000 |
| | Metaproterenol sulfate orciprenaline sulfate | Beta-adrenergic agonist | 0.569 | 0.034 | 0.0217 |
| | Bosentan | endothelin-1 inhibition | 0.576 | 0.029 | 0.0085 |
| | Trazodone hydrochloride | 5-HT uptake inhibitor | 0.577 | 0.019 | 0.0015 |
| | Salbutamol | Beta adrenergic agonist | 0.577 | 0.008 | 0.0000 |
| | Dipivefrin hydrochloride | Prodrug, epinephrine produced is an adrenergic agonist | 0.586 | 0.012 | 0.0001 |
| | Ebselen | Cyclooxygenase inhibitor | 0.590 | 0.023 | 0.0023 |
| | Tulobuterol | beta-2-adrenergic receptor agonist | 0.599 | 0.022 | 0.0012 |
| | Terbutaline hemisulfate | Beta-2 adrenergic agonist | 0.610 | 0.023 | 0.0012 |
| | Racepinephrine HCl | non-selective agonist of all adrenergic receptors | 0.616 | 0.013 | 0.0001 |
| | Formoterol fumarate | ß2-agonist | 0.617 | 0.018 | 0.0003 |
| 100nM negative hits | Levonordefrin | Adrenergic receptor agonist | 0.536 | 0.016 | 0.0023 |
| | Fenoterol hydrobromide | Beta adrenergic agonist | 0.543 | 0.020 | 0.0050 |
| | Benzethonium chloride | Detergent | 0.552 | 0.021 | 0.0039 |
| | Dipivefrin hydrochloride | Prodrug, epinephrine produced is an adrenergic agonist | 0.555 | 0.023 | 0.0051 |
| | Salbutamol | Beta adrenergic agonist | 0.570 | 0.014 | 0.0003 |
| | Isoetharine mesylate salt | Beta adrenergic agonist | 0.577 | 0.022 | 0.0018 |
| | Bosentan | endothelin-1 inhibition | 0.579 | 0.015 | 0.0003 |
| | Racepinephrine HCl | non-selective agonist of all adrenergic receptors | 0.595 | 0.027 | 0.0026 |
