# Supplementary material for: Dopamine receptor antagonists as potential therapeutic agents for ADPKD
Source: PLoS One. 2019 May 6;14(5):e0216220. doi: 10.1371/journal.pone.0216220 (PMC6502331; doi:10.1371/journal.pone.0216220)
Supplement: S3 Table — Measurements made in P43 mice. (PDF) [file pone.0216220.s008.pdf]

S3 Table. Effect of Domperidone treatment on body weight (BW), kidney weight (KW) and number of glomerular cyst in long term treatment group. Measurements made in P43 mice.

	WT	***Pkd1***$^{-/-}$ **DMSO treated**	***Pkd1***$^{-/-}$ **Domperidone treated**
**BW (gm)**	17 ± 4.04	8± 0	16 ± 3.51
**KW/BW (%)**	0.2 ± 0	1.35 ± 0.49	1 ± 0.50
**Glomerular cyst**	0 ± 0	66 ± 2.82	21 ± 32.96
