# Supplementary material for: Dopamine receptor antagonists as potential therapeutic agents for ADPKD
Source: PLoS One. 2019 May 6;14(5):e0216220. doi: 10.1371/journal.pone.0216220 (PMC6502331; doi:10.1371/journal.pone.0216220)
Supplement: S2 File — (PDF) [file pone.0216220.s011.pdf]

Full unedited gel for Fig 2

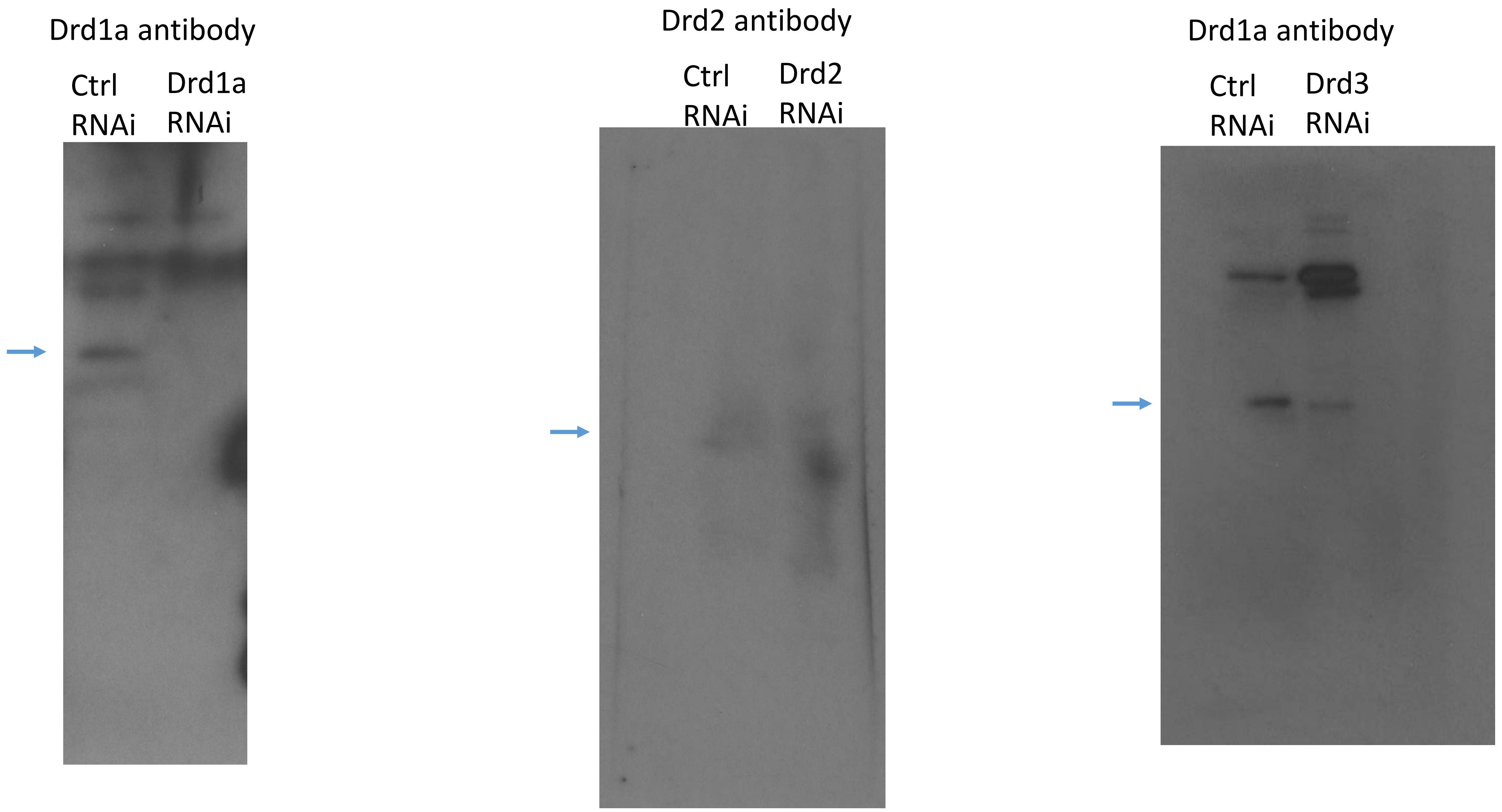

Drrd1a antibody

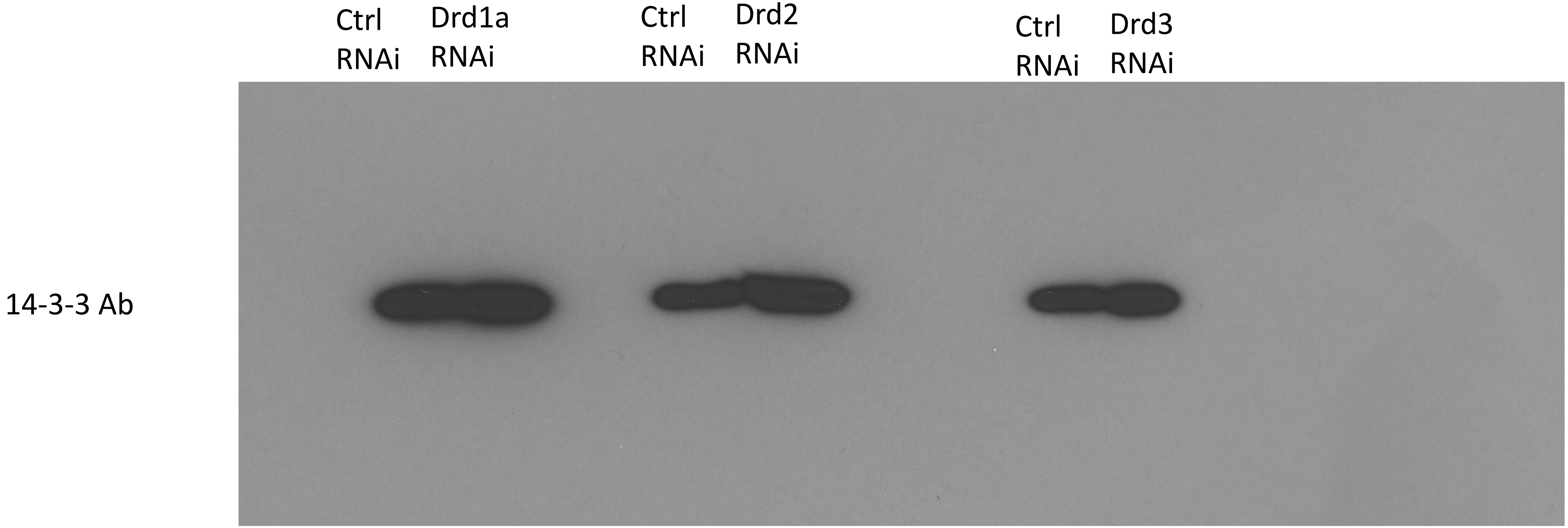

Ctrl RNAi
Drd1a RNAi
Ctrl RNAi
Drd2 RNAi
Ctrl RNAi
Drd3 RNAi
14-3-3 Ab

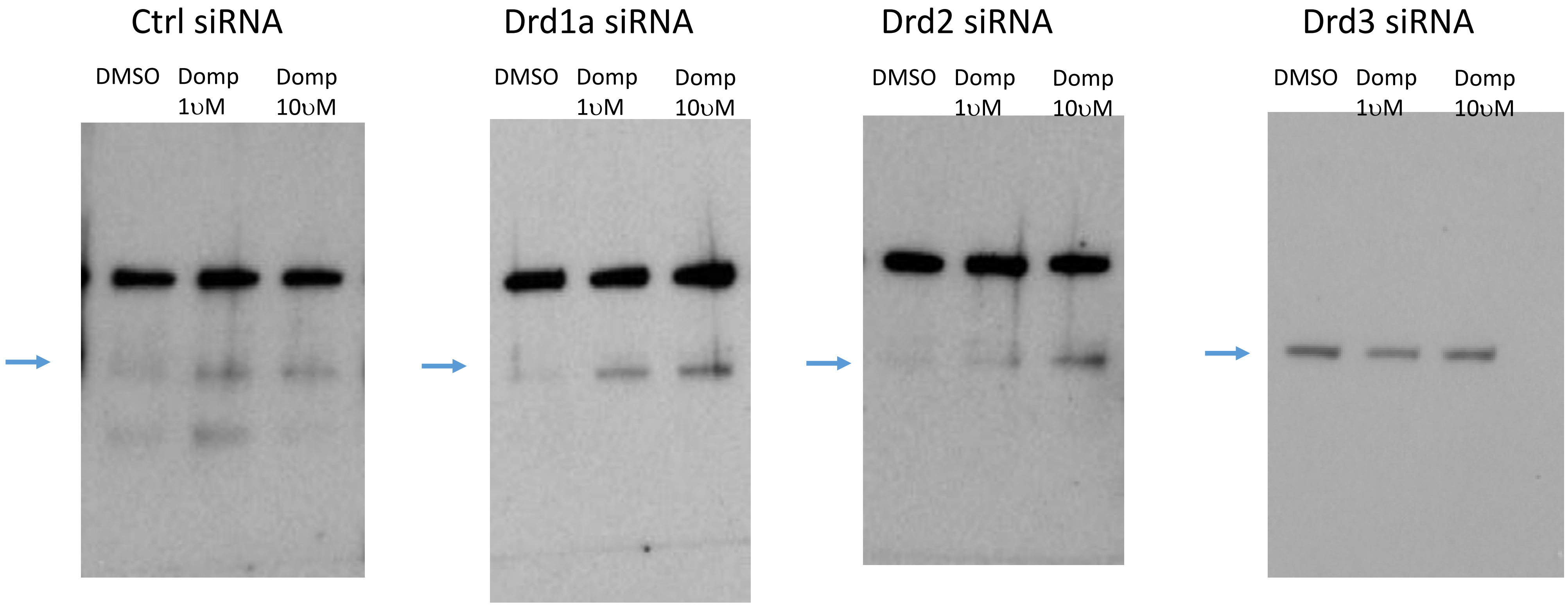
Ctrl siRNA
DMSO
Domp 1υM
Domp 10υM
Drd1a siRNA
DMSO
Domp 1υM
Domp 10υM
Drd2 siRNA
DMSO
Domp 1υM
Domp 10υM
Drd3 siRNA
DMSO
Domp 1υM
Domp 10υM

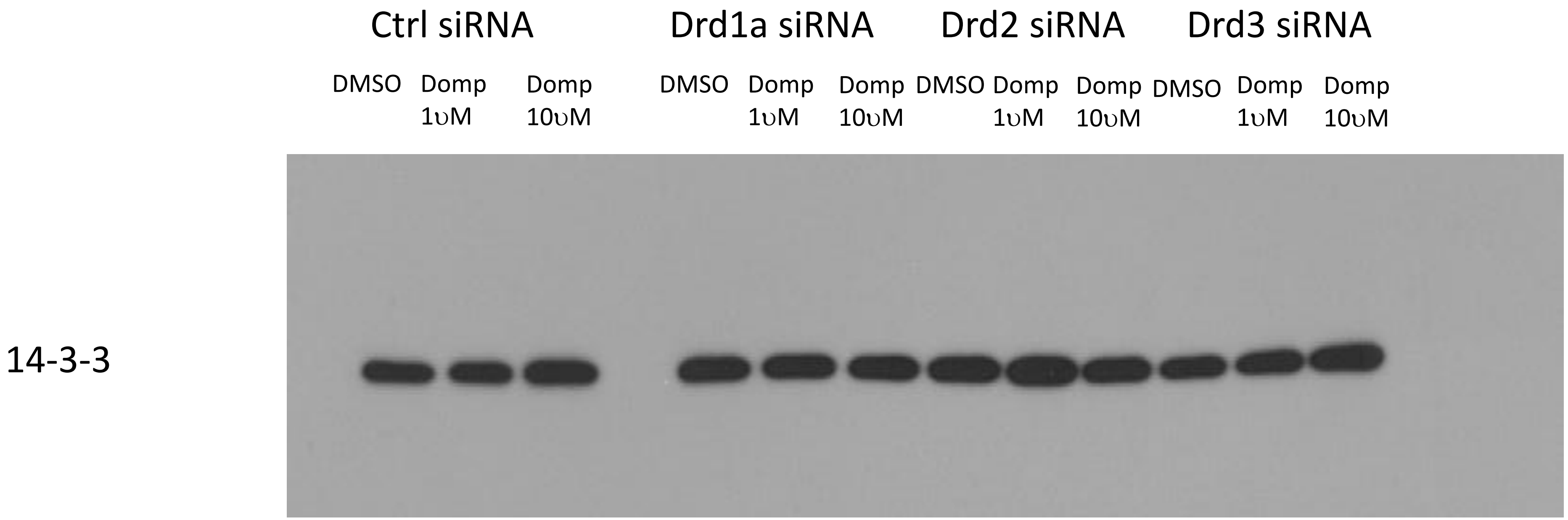
Ctrl siRNA
Drd1a siRNA
Drd2 siRNA
Drd3 siRNA
DMSO
Domp 1υM
Domp 10υM
DMSO
Domp 1υM
Domp 10υM
DMSO
Domp 1υM
Domp 10υM
DMSO
Domp 1υM
Domp 10υM
14-3-3
