# Supplementary material for: Dopamine receptor antagonists as potential therapeutic agents for ADPKD
Source: PLoS One. 2019 May 6;14(5):e0216220. doi: 10.1371/journal.pone.0216220 (PMC6502331; doi:10.1371/journal.pone.0216220)
Supplement: S1 Data — (PDF) [file pone.0216220.s012.pdf]

Data for Fig 1C

HDAC-GFP-PKD1 loxp/loxP

DMSO Total number of cell

	Image 1	Image 2	Image 3	Image 4	Image 5
Nuclear	55	63	90	110	78
Both	3	5	1	8	4
Cytoplasm	1	0	3	14	12

Domperidone 10nM

	Image 1	Image 2	Image 3	Image 4	Image 5	Image 6
Nuclear	32	70	61	67	49	57
Both	11	26	11	20	14	19
Cytoplasm	21	12	4	7	34	19

Domperidone 100nM

	Image 1	Image 2	Image 3	Image 4	Image 5	Image 6
Nuclear	6	5	8	6	1	4
Both	18	15	19	36	25	20
Cytoplasm	110	102	59	90	76	78

Domperidone 1µM

	Image 1	Image 2	Image 3	Image 4
Nuclear	3	3	1	6
Both	9	7	3	7
Cytoplasm	84	141	100	143

Loxapine

	Image 1	Image 2	Image 3	Image 4	Image 5
Nuclear	4	0	8	4	12
Both	26	10	32	32	30
Cytoplasm	90	77	84	68	103

PKD1 null transiently transfected with HDAC5-GFP

DMSO

	Image 1	Image 2	Image 3	Image 4
Nuclear	98	122	109	120
Cytoplasm	4	8	12	7

Domperidone

	Image 1	Image 2	Image 3	Image 4
Nuclear	7	25	17	10
Cytoplasm	93	140	66	78

Loxapine

	Image 1	Image 2	Image 3	Image 4
Nuclear	0	6	10	15
Cytoplasm	30	150	62	105

Data for Fig 2B

HDAC-GFP-PKD1 loxp/loxP

DMSO Total number of cell

	Image 1	Image 2	Image 3	Image 4	Image 5
Nuclear	104	90	111	81	124
Both	20	17	6	7	8
Cytoplasm	3	7	10	0	4

Spiperone

	Image 1	Image 2	Image 3
Nuclear	5	7	5
Both	6	5	10
Cytoplasm	129	98	119

Haloperidol

	Image 1	Image 2	Image 3
Nuclear	11	6	20
Both	3	10	10
Cytoplasm	115	121	59

Raclopride

	Image 1	Image 2	Image 3	Image 4	Image 5	Image 6	Image 7
Nuclear	24	30	37	36	38	41	33
Both	32	24	23	28	19	32	44
Cytoplasm	16	28	30	22	21	28	32

SCH23390

	Image 1	Image 2
Nuclear	110	90
Both	2	5
Cytoplasm	4	4

Data for Fig 3G

HDAC-GFP-PKD1 loxp/loxP

DMSO Total number of cell

	Image 1	Image 2	Image 3	Image 4	Image 5
Nuclear	104	90	111	81	124
Both	20	17	6	7	8
Cytoplasm	3	7	10	0	4

Oka 1µM

	Image 1	Image 2	Image 3
Nuclear	2	0	0
Both	1	0	10
Cyto	110	105	130

Tautomycetin 5µM

	Image 1	Image 2
Nuclear	101	109
Both	0	0
Cyto	5	6

H-89 50µM

	Image 1	Image 2	Image 3
Nuclear	12	18	19
Both	2	7	9
Cyto	110	71	90

Oka 100nM washout DMSO

	Image 1	Image 2	Image 3
Nuclear	90	100	120
Both	10	2	2
Cyto	0	2	14

Oka 100nM washout H-89 30µM

	Image 1	Image 2	Image 3
Nuclear	2	2	7
Both	0	6	4
Cyto	118	109	110

Fsk

	Image 1	Image 2	Image 3
Nuclear	140	99	132
Both	0	0	1
Cyto	1	0	4

Fsk+Oka 1µM

	Image 1	Image 2	Image 3
Nuclear	13	52	45
Both	20	0	12
Cyto	71	30	20

Fsk+Tautomycetin 5µM

	Image 1	Image 2
Nuclear	102	110
Both	7	0
Cyto	0	2

Fsk+H-89 50µM

	Image 1	Image 2	Image 3
Nuclear	140	80	95
Both	0	0	1
Cyto	2	0	0

Domperidone

	Image 1	Image 2	Image 3	Image 4	Image 5
Nuclear	10	12	5	2	6
Both	21	34	30	22	43
Cyto	64	54	67	75	80

50µM GO6983+Domperidone

	Image 1	Image 2
Nuclear	6	13
Both	4	11
Cyto	110	95

PKD1 null transiently transfected with HDAC5-GFP

DMSO

	Image 1	Image 2	Image 3	Image 4
Nuclear	98	122	109	120
Cytoplasm	4	8	12	7

Oka 1µM

	Image 1	Image 2
Nuclear	40	36
Cytoplasm	70	80

Tautomycetin 5µm

	Image 1	Image 2	Image 3
Nuclear	79	100	125
Cytoplasm	2	9	12

Data for Fig 4C

DMSO

Normalized % cystic area

0.719665032	1.08274781
1.048543279	1.149043879
1.412023751	1.004401425
0.775745773	0.807829051
0.894532003	
1.172379239	0.933088758
1.241971	0.758029

Domperidone

Normalized cyst area

0.27454192	0.340603786
0.771160953	0.79208397
0.724396359	0.604770903
	0.457404195
0.734092869	0.842410023
0.687117199	0.637076204
0.772500001	0.60694026
1.241971416	

Data for Fig 4F

DMSO

Normalized fraction of Ki67 nuclei

1.128997761
0.871002239
0.760924687
1.239075313
1.167746657
0.832253343
1
1

Domperidone

Normalized fraction of Ki67 nuclei

0.638772328
0.91497046
1.017544133
0.973928436
0.766377031
0.180382056
0.438250141
0.897066014

0.509678838

Data for Fig 5C

DMSO

Normalized % cystic area
0.985649835
1.033451161
1.021706492
0.959192512

Domperidone

Normalized % cystic area
0.696379704
0.645533752

Data for S1C Fig

Pkd1-/-HDAC5+/+

Normalized % cystic area

0.89368111	1.106319
0.921257764	1.078742
1.023967894	0.994305
0.981727503	

Pkd1-/-HDAC5+/-

Normalized % cystic area

0.43706151	0.542078
0.721545792	0.577819
1.015624448	1.278841
0.50560219	0.829788
0.665140407	0.538029
0.648682542	0.813311
0.939999313	0.864131
0.214958616	0.130897
0.266063837	0.281869
0.177668229	0.107571
0.137102521	0.144013

Data for S1D Fig

Pkd1-/-HDAC5+/+ P21

Normalized BUN

1.816729992
0.546603231
0.636666777

Pkd1-/-HDAC5+/- P21

Normalized BUN

1.372887826
1.799517514
0.591475562
0.886656721
0.545892022
1.304420799
0.647081004
0.448122636
0.68718355
1.139347528

Pkd1-/-HDAC5+/+ P28

Normalized BUN

0.924243438

0.836483525
0.958527846
1.098932149

Pkd1-/-HDAC5+/- P28

Normalized BUN

0.744296549
0.660978358
0.770085526

Data for S2C Fig & S2D Fig

Antagonist or agonist in positive hits

	Fraction nuclear	SEM
DMSO	0.456	0.002
I3A	0.240	0.004
PMA	0.209	0.004
Cisapride	0.240	0.023
Loxapine succinate	0.270	0.036
Oxybutynin chloride	0.277	0.017
Cyproheptadine hydrochloride	0.278	0.026
Domperidone	0.281	0.041
Pimozide	0.309	0.015
Pizotifen malate	0.315	0.022
Pimethixene maleate	0.323	0.032
Tridihexethyl chloride	0.333	0.013
Deptropine citrate	0.338	0.040
Amitryptiline hydrochloride	0.346	0.023
Diphemanil methylsulfate	0.353	0.012
Clebopride maleate	0.355	0.024
Tetrahydrozoline hydrochloride	0.357	0.022
Beta-Escin	0.359	0.027
Methyldopate hydrochloride	0.359	0.027
Lofexidine	0.360	0.025
Flunisolide	0.363	0.016
Tizanidine	0.333	0.012

Antagonist or agonist in negative hits

	Fraction nuclear	SEM
DMSO	0.456	0.002
I3A	0.240	0.004
PMA	0.209	0.004
(+)-Isoproterenol (+)-bitartrate salt	0.550	0.038
Alprostadil	0.553	0.035
Ritodrine hydrochloride	0.553	0.029
Pridinol methanesulfonate salt	0.556	0.030
Fenoterol hydrobromide	0.559	0.039
Nylidrin	0.566	0.026
Levonordefrin	0.569	0.008
Metaproterenol sulfate□ orciprenaline sulfate	0.569	0.034
Salbutamol	0.577	0.008
Dipivefrin hydrochloride	0.586	0.012
Tulobuterol	0.599	0.022
Terbutaline hemisulfate	0.610	0.023
Racepinephrine HCl	0.616	0.013
Formoterol fumarate	0.617	0.018
Levonordefrin	0.536	0.016
Fenoterol hydrobromide	0.543	0.020
Salbutamol	0.570	0.014
Isoetharine mesylate salt	0.577	0.022
Racepinephrine HCl	0.595	0.027

Prazosin hydrochloride	0.339	0.022
Quipazine dimaleate salt	0.342	0.034
Atropine sulfate monohydrate	0.343	0.020
(S)-(-)-Atenolol	0.347	0.021
Amiodarone hydrochloride	0.349	0.021
Buflomedil hydrochloride	0.353	0.024
SR-95639A dihydrochloride	0.354	0.012
Raclopride	0.355	0.019
Chlorprothixene hydrochloride	0.356	0.012

Data for S3C Fig

HDAC-GFP-PKD1 loxp/loxP

DMSO Total number of cell

	Image 1	Image 2	Image 3	Image 4	Image 5
Nuclear	104	90	111	81	124
Both	20	17	6	7	8
Cytoplasm	3	7	10	0	4

Flow-DMSO

	Image 1	Image 2	Image 3	Image 4	Image 5	Image 6
Nuclear	37	15	14	9	17	8
Both	136	140	59	82	84	50
Cytoplasm	44	66	31	18	12	18

Flow-terbutaline

	Image 1	Image 2	Image 3
Nuclear	62	58	77
Both	1	3	12
Cytoplasm	2	5	10

PMA-DMSO

	Image 1	Image 2
Nuclear	1	1
Both	24	25
Cytoplasm	125	98

PMA-Terbutaline

	Image 1	Image 2	Image 3
Nuclear	25	44	54
Both	22	17	20
Cytoplasm	10	14	21

PKD1 loxp/loxP transiently tranfected with HDAC5-GFP

DMSO

	Image 1	Image 2
Nuclear	13	12
Both	2	1
Cytoplasm	0	1

Domperidone

	Image 1	Image 2	Image 3	Image 4
Nuclear	1	1	1	1
Both	2	7	0	1

Cytoplasm	3	12	4	15

Data for S3C Fig

Kif3a-/- transientky transfected with HDAC5-GFP

DMSO Total number of cell

	Image 1	Image 2	Image 3
Nuclear	96	70	80
Cytoplasm	11	5	11

Domperidone

	Image 1	Image 2	Image 3
Nuclear	14	5	2
Cytoplasm	112	90	100

Loxapine

	Image 1	Image 2	Image 3
Nuclear	10	1	5
Cytoplasm	120	110	81

HDAC-GFP-PKD1 loxp/loxP

Raclopride 10nM

	Image 1	Image 2	Image 3	Image 4
Nuclear	53	50	35	38
Both	33	33	27	31
Cytoplasm	27	50	16	19

Data for S3 Table

Genotype	Treatment	% Glomerular cyst	Body Weight (gm)	Kidney Weight (mg)	
WT	DMSO	Not calculated	14	17	15
WT	DMSO	Not calculated	22	18	17
WT	Domperidone	0	17	16	16
Pkd1 null	DMSO	68	8	41	39
Pkd1 null	DMSO	64	8	75	57
Pkd1 null	Domperidone	0	20	39	36
Pkd1 null	Domperidone	59	13	87	91
Pkd1 null	Domperidone	4	16	75	87
